# Alternative Splicing-Based Differences Between Hepatocellular Carcinoma and Intrahepatic Cholangiocarcinoma: Genes, Immune Microenvironment, and Survival Prognosis

**DOI:** 10.3389/fonc.2021.731993

**Published:** 2021-10-25

**Authors:** Dingan Luo, Deze Zhao, Mao Zhang, Chuan Hu, Haoran Li, Shun Zhang, Xiaowu Chen, Lakshmi Huttad, Bailiang Li, Cheng Jin, Changwei Lin, Bing Han

**Affiliations:** ^1^ Department of Hepatobiliary and Pancreatic Surgery, The Affiliated Hospital of Qingdao University, Qingdao, China; ^2^ Department of Thoracic Surgery, Xiangya Hospital, Central South University, Changsha, China; ^3^ Medical College, Qingdao University, Qingdao, China; ^4^ Asian Liver Center, Department of Surgery, Medical School of Stanford University, Stanford, CA, United States; ^5^ Department of Radiation Oncology, Medical School of Stanford University, Stanford, CA, United States; ^6^ Department of Gastrointestinal Surgery, The Third Xiangya Hospital of Central South University, Changsha, China

**Keywords:** hepatocellular carcinoma, intrahepatic cholangiocarcinoma, alternative splicing, immune, prognostic models

## Abstract

Alternative splicing (AS) event is a novel biomarker of tumor tumorigenesis and progression. However, the comprehensive analysis of hepatocellular carcinoma (HCC) and intrahepatic cholangiocarcinoma (ICC) is lacking. Differentially expressed analysis was used to identify the differentially expressed alternative splicing (DEAS) events between HCC or ICC tissues and their normal tissues. The correlation between DEAS events and functional analyses or immune features was evaluated. The cluster analysis based on DEAS can accurately reflect the differences in the immune microenvironment between HCC and ICC. Forty-five immune checkpoints and 23 immune features were considered statistically significant in HCC, while only seven immune checkpoints and one immune feature in ICC. Then, the prognostic value of DEAS events was studied, and two transcripts with different basic cell functions (proliferation, cell cycle, invasion, and migration) were produced by *ADHFE1* through alternative splicing. Moreover, four nomograms were established in conjunction with relevant clinicopathological factors. Finally, we found two most significant splicing factors and further showed their protein crystal structure. The joint analysis of the AS events in HCC and ICC revealed novel insights into immune features and clinical prognosis, which might provide positive implications in HCC and ICC treatment.

## Introduction

**Figure 1:**
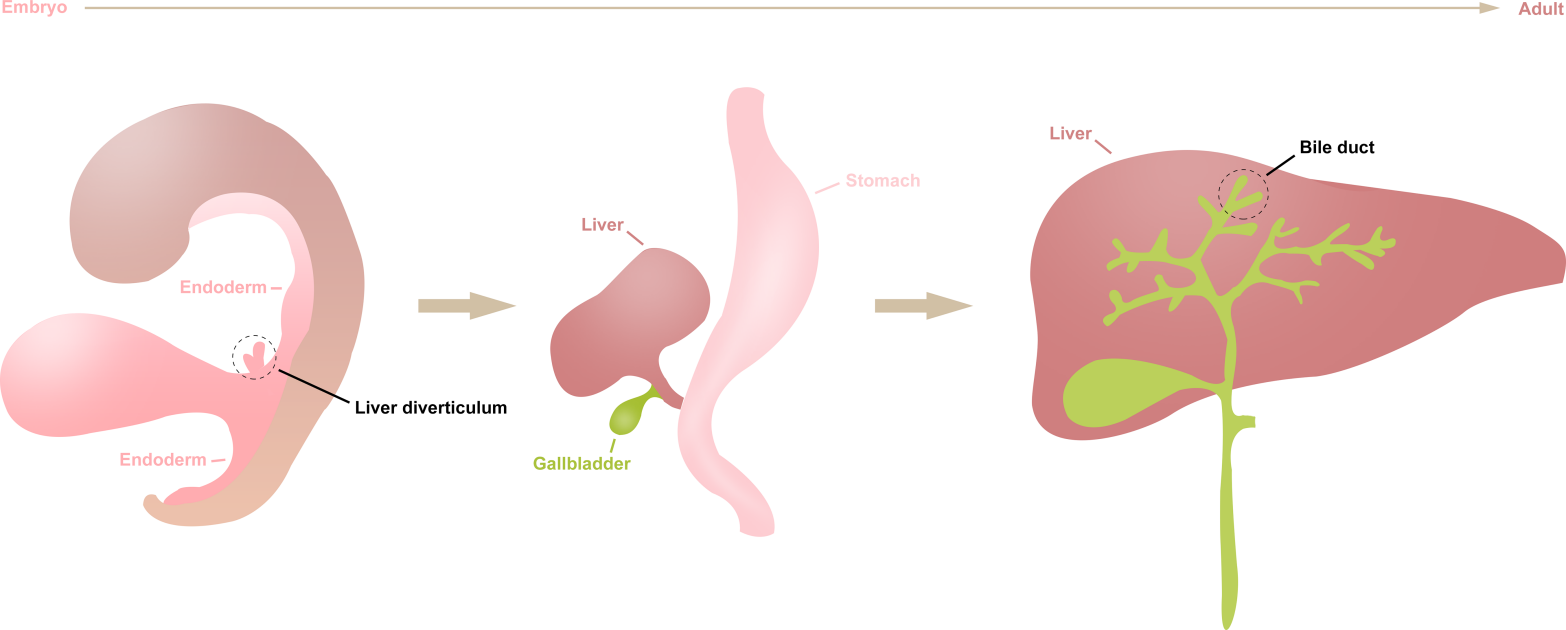
The differentiation of liver diverticulum. The liver and intrahepatic bile duct arise from the liver diverticulum of the endoderm during early embryogenesis, then gradually differentiate into mature organs during human growth and development.

Primary carcinoma of the liver (PCL) is a common tumor of digestive system (1). As the 5-year survival rate is not optimistic, ranging from 5% to 30%, researchers have made great efforts to explore the prevention, diagnosis, and treatment of PCL in recent years (2). Generally, histological subtypes of PCL can be divided in hepatocellular carcinoma (HCC), intrahepatic cholangiocarcinoma (ICC), combined hepatocellular cholangiocarcinoma (CHCC), and others, of which the HCC and ICC are the two most common subtypes in all histological types of PCL, accounting for 70–80% and 7–10% of PCL, respectively (3, 4). Since the liver and bile duct share similar endodermal developmental origins (**Figure 1**), HCC and ICC have many similar genomic and other molecular characteristics changes during their development (5). However, studies have shown there are also many tumor heterogeneities between them, such as differences in epidemiology and prognosis (6). Therefore, it is imperative to investigate the similarities and differences between HCC and ICC to achieve accurate treatment for a wide range of patients.

Alternative splicing (AS) is a critical step in the post-transcriptional modification of mRNA. Mature mRNAs with different structures and functions can be produced by acting on pre-RNA by seven types of splicing. Therefore, despite the limited number of human genes, the presence of AS events increases protein diversity and cellular complexity (7). In recent years, it has been confirmed that AS are closely related to a variety of tumor signaling pathways, including sustaining proliferative signaling, evading growth suppressors, angiogenesis, vascular invasion, and metastasis (8). Recently, some studies have reported that AS events can be used as factors to predict the prognosis and recurrence of HCC or ICC, respectively (9–11). More importantly, many evidence demonstrate that AS affects the formation of the immune microenvironment through various pathways (12, 13). However, there are no studies exploring the differences of immune features or clinical prognosis between HCC and ICC based on AS patterns.

Here, we identified tumor-specific splicing events based on the Cancer Genome Atlas (TCGA) data portal and explored the potential biological functions of them. Subsequently, due to the interest in the formation of the immune microenvironment, the correlation between AS events and immune features was also studied in HCC and ICC. In addition, we studied the impact of AS events on the prognosis and established four nomograms based on AS and clinicopathological factors. Further, we focused on AA_ADHFE1, an AS event that affects both OS and DFS, and verified its cell biological function *in vitro*, and predicted the potential splicing factors that affect its production. This article provides important guidance for the following research on AS in HCC and ICC.

## Materials and Methods

### Data Acquisition and Processing

The selection criteria for this study are as follows: (1) definite histological diagnosis of HCC and ICC; (2) definitive clinical data; (3) at least 30 days of overall survival after initial pathologic diagnosis (14–16); (4) complete RNA-sequencing data. Gene expression quantification data and related clinical data of the HCC and ICC were downloaded from TCGA database, and DESeq2 package was used to normalize the data portal (17, 18). Besides, the Percent Spliced In (PSI) value, which is a widely accepted indicator to quantify the AS events, was downloaded from TCGA SpliceSeq (19). To obtain the most reliable AS events set, we set a series of strict filter conditions (Percentage of samples with PSI value more than 0.75, average of PSI value more than 0.05) (20). UpSet plots were generated by the package of UpSetR (version 1.4.0) to display interactive sets between each types of AS events (21). In addition, Circos plots were generated by the software of Circos (version 0.69-6) to depict the details of splicing events and location of parent gene in whole chromosome (22).

### Identification of DEAS Events and Potential Functional Analyses in HCC and ICC

To identify the tumor-specific splicing events between tumor tissue and normal tissue, the PSI value of patients was calculated (including 343 HCC tissues and 48 normal tissues, 33 ICC tissues and 8 normal tissues). Benjamini & Hochberg (BH) correction was used to adjust p-values. The AS events with the adj.p < 0.05 and|log2FC|>1 were considered to be significantly upregulated or downregulated. And the Venn diagram was developed to represent the differences between differentially expressed alternative splicing (DEAS) events and differentially expressed genetic (DEG). The parent genes of DEAS event were submitted to the String 11.0 online database for protein-protein interaction (PPI) analysis (23). The relationship network was then illustrated by Cytoscape (24). In addition, these parent genes were also used as the Gene Ontology (GO), Kyoto Encyclopedia of Genes and Genomes (KEGG) pathway analyses *via* Metascape.

### Analyses of Immune Characteristics in HCC and ICC

Additionally, the “ConsensusClusterPlus” package was performed to classify patients based on the DEAS events (25). Subsequently, immune features were analyzed using ESTIMATE (26) and ssGSEA (27). The correlation analysis was conducted to clarify the relationship between DEAS clusters (or two cancer types) and immune characteristics (ESTIMATE Score, Stromal Score, Tumor Purity, Immune Score, Cytolytic activity, NK cells, etc.).

### The Effect of Alternative Splicing on the Prognosis of HCC and ICC

To standardize the PSI data, the median PSI value was used as a threshold to divide patients into two groups for each AS event. Univariate cox analysis and LASSO (alignment=lambda, nfold=10, gamma = c(0, 0.25, 0.5, 0.75, 1) analysis were used to identify potential prognostic factors (28). Later, the multivariate cox analysis was used to identify the independent risk factors for overall survival (OS) and disease-free survival (DFS) (named OS-DEAS events and DFS-DEAS events). And the risk scores of patients were calculated based on multivariate cox model (named OS-model and DFS-model, respectively).

Then, to combine the OS- or DFS-model with clinicopathological data, the nomograms were developed by rms package (v6.2.0) (29, 30). In addition, the area under the curve (AUC) of the receiver operating characteristic (ROC) and the consistency index (C-index) was calculated to evaluate the predictive ability of nomogram or other models (31).

### Functional Verification of AA_ADHFE1 in Cell Lines of HCC

The selection and cultivation of cell lines, the materials and methods of functional experiments, the collection and processing of tissue samples, and other methods can be found in the supplementary materials.

### Correlation Analyses Between SFs and OS-/DFS-DEAS Events in HCC and ICC

The data of splicing factors (SFs) which were validated in previous studies were downloaded from the SpliceAid 2 database (32). In addition, the expressions of SFs were downloaded from TCGA database, and the DESeq2 package was used to normalize the data portal (18). Correlation analyses were performed to determine the potential regulatory relationship between both OS- or DFS-DEAS events and SFs. In addition, crystal structures of SFs are obtained from Protein Databank.

### Statistical Analysis

All statistical analyses were conducted by R software (version 3.6.1) (24). Categorical data were performed using chi-square (χ2) test. Spearman’s rank correlation analysis was utilized for non-normal distribution data. Student’s t-test and ANOVA test were utilized to compare continuous variables. Survival curves were compared using log-rank test and performed using the Kaplan–Meier method. Pearson correlation was utilized for continuous variables that meet normal distribution. The results of Cox analysis were presented as the mean ± S.D., and P<0.05 (two-tailed) was considered statistically significant.

## Results

### Overview of AS Events in HCC and ICC

**Figure 2:**
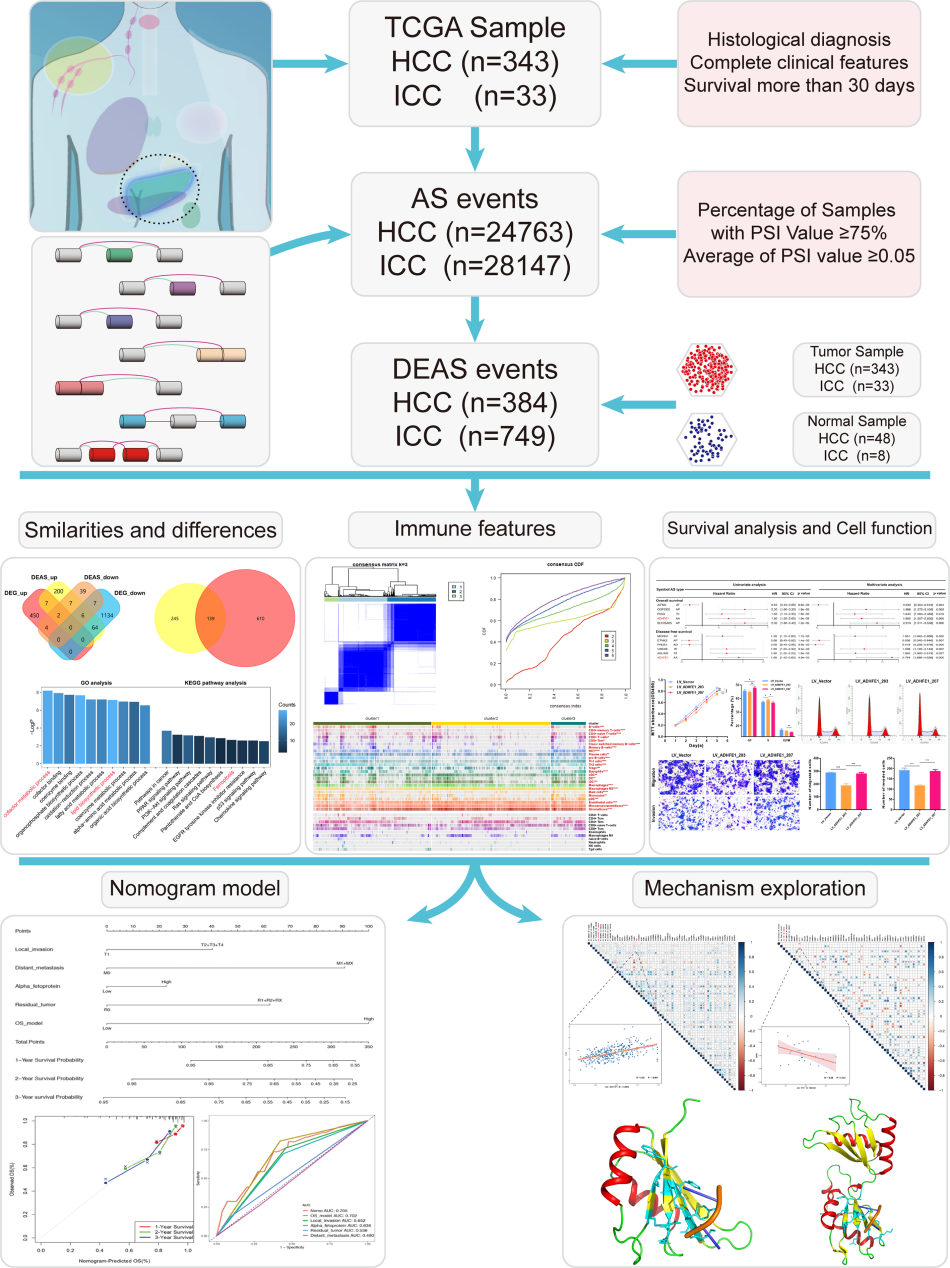
The flowchart of the present study.

The pipeline of our research is shown in **Figure 2**. A total of 343 HCC patients and 33 ICC patients were included in this study (the baseline characteristic of patients is listed in **Supplementary Tables S1**, **S2**). Subsequently, 24,763 AS events in 8,434 genes were further detected in 343 HCC patients, and 28,147 AS events and 8,094 genes were detected in 33 ICC patients. These data indicated that one gene could have nearly three types of AS events. AS events include seven subtypes (**Supplementary Figure S1A**), which were all detected both in HCC and ICC (**Supplementary Figures S1B, C**), and ES was the most common AS type and ME is the rare type of AS in tumors. As shown in **Supplementary Figures S1D, E**, the UpSet plots of HCC and ICC showed the sets of each AS type. Moreover, two Circos-plots were developed to depict the details of AS events and location of parent gene in whole chromosome (**Supplementary Figures S1F, G**). The above results indicate that alternative splicing, which leads to the different arrangements and combinations of exons and introns, is responsible for the diversity of the transcriptome.

### Identification of DEAS Events and Potential Functional Analyses in HCC and ICC

To identify the tumor-specific splicing events in tumor tissues and normal tissues, the comparison of PSI value between these tissues was performed. Finally, 384 DEAS events were identified from 336 genes in HCC. Meanwhile, in ICC, 749 DEAS events were found from 622 genes in ICC. The details of DEAS events are shown in **Supplementary Tables S3**, **S4**. Intriguingly, we found the AP type was the predominant DEAS mode in both HCC and ICC, and the significant differences in the distribution of seven splicing modes about DEAS events between HCC and ICC (**Supplementary Figure S2A**).

**Figure 3:**
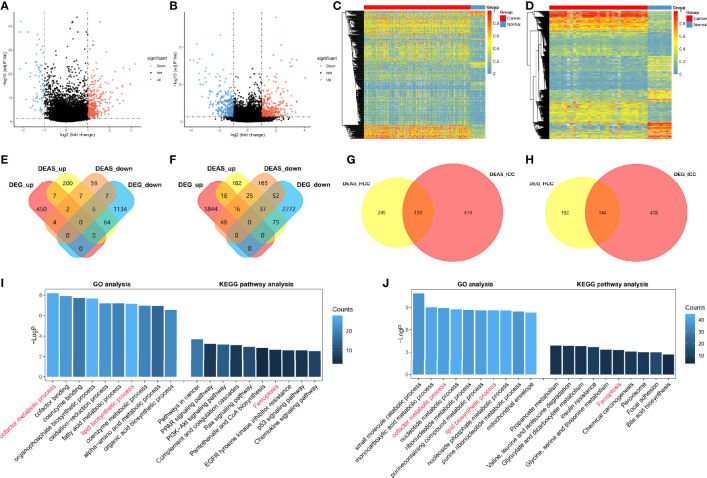
Identification of DEAS events and potential functional analyses in HCC and ICC. **(A, B)** The tumor-specific AS events between tumor tissues and normal tissues. DEAS events identified in HCC **(A)** and ICC **(B)**. **(C, D)** Heatmaps of the DEAS events in HCC **(C)** and ICC **(D)**, respectively. **(E, F)** Two Venn diagrams showed the common of DEAS events (yellow and orange) and DEG (red and blue) among HCC **(E)** and ICC **(F)**. **(G, H)** Two Venn diagrams were generated to show the common of DEAS events **(G)** and DEG **(H)** between HCC (yellow) and ICC (red). **(I, J)** GO, KEGG pathway etc. analyses of DEAS events in HCC **(I)** and ICC **(J)**; the x axis represents the annotations of GO or KEGG pathway etc.; the y axis reflects the number of corresponding genes.

After the DEAS events of HCC and ICC were identified and the upregulation and downregulation DEAS events were displayed in the volcano plots (**Figures 3A, B**), unsupervised hierarchical consensus clustering was performed basing on DEAS events. The results showed that samples of cancer and normal tissues can be clearly separated into two groups, which means that the DEAS events identified above were convincing (**Figures 3C, D**). Moreover, we developed two Venn diagrams to depict the relationship of DEAS events and DEG in HCC (**Figure 3E**) or ICC (**Figure 3F**), and the details are shown in **Supplementary Tables S5**, **S6**. Intriguingly, whether in HCC or ICC, many genes (such as ADRA1A and KIF4A) displayed some opposite features of AS events in tumor and normal tissues (**Supplementary Figure S2B**). Moreover, 139 AS events and 144 DEG were identified as common DEAS events and DEG between HCC (**Figure 3G**) and ICC (**Figure 3H**).

Subsequently, the corresponding proteins of DEAS events were used to construct PPI networks for HCC (**Supplementary Figure S3A**) and ICC (**Supplementary Figure S3B**), and the PPI networks analyses demonstrated their interactive relationship in normal condition. We further filtered 10 hub genes from the protein-protein interaction network in HCC (**Supplementary Figure S3C**) and ICC (**Supplementary Figure S3D**), respectively. Two hub genes, Fibronectin 1 (FN1) and Serpin peptidase inhibitor clade A member 1 (SERPINA1), were identified as common genes between HCC and ICC, but other eight hub genes are different. Moreover, we analyzed the potential functions of DEAS events by GO and KEGG pathway (**Figures 3I, J**). The results suggested that GO categories related to the metabolic process, including “cofactor metabolic process” in HCC and “small molecule catabolic process” in ICC. And “cofactor metabolic process” and “lipid biosynthetic process” in GO analysis were identified as common metabolic processes between HCC and ICC. Moreover, KEGG pathways enriched were associated with tumorigenesis, such as “PI3K-Akt signaling pathway” in HCC and “Chemical carcinogenesis” in ICC. And only the “Ferroptosis” was confirmed as common KEGG pathway between HCC and ICC. Intriguingly, immune-related pathways were also enriched in HCC (not in ICC), such as “Complement and coagulation cascades” and “chemokine signaling pathway,” which indicated that DEAS events may be involved in immune microenvironment formation in HCC patients. These results prove that HCC and ICC are partly common in DEAS events and potential functional, and they may provide a reference for the study of CHCC. More importantly, more DEAS events (86%) and pathways (90%) are different between HCC and ICC.

### DEAS Clusters and Immune Features in HCC or ICC

**Figure 4:**
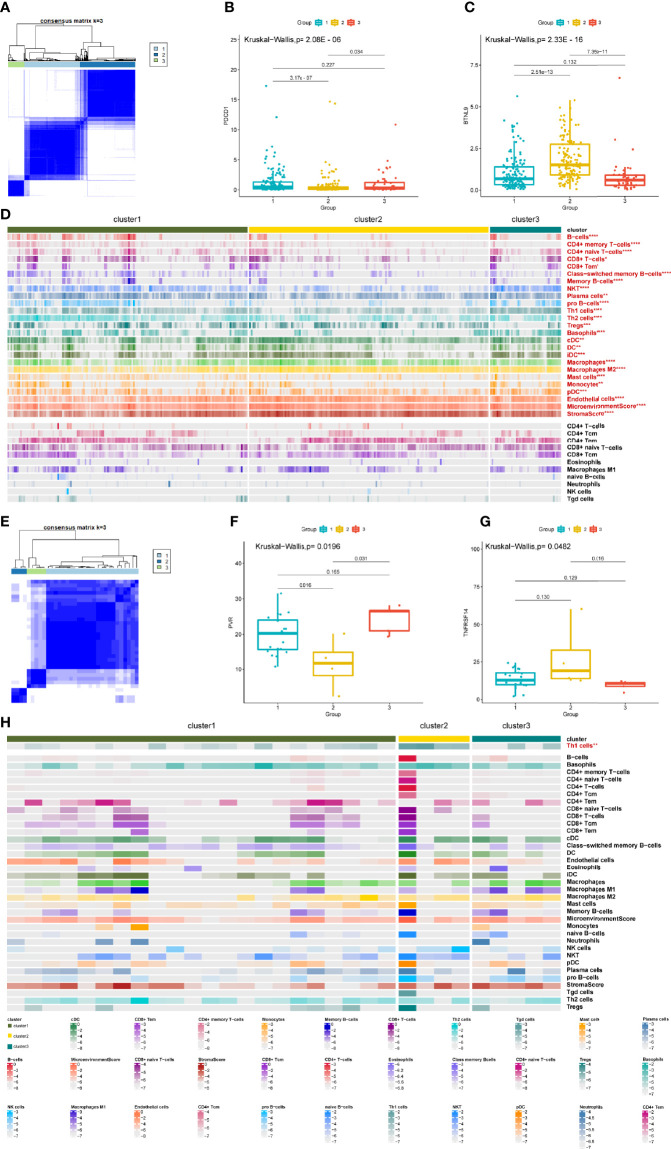
DEAS clusters correlated with immune features in HCC and ICC. **(A)** Consensus matrix heatmap of HCC. **(B, C)** The compassion of two representative immune checkpoints in three clusters. **(D)** Heatmap of the DEAS events in HCC ordered by clusters. **(E)** Consensus matrix heatmap of ICC. **(F, G)** The compassion of two representative immune checkpoints in three clusters. **(H)** Heatmap of the DEAS events in ICC ordered by clusters, with annotations related with each cluster. *P < 0.05, **P < 0.01, ***P < 0.001, ****P < 0.0001.

Immune microenvironment is crucial to the development and recrudescence of tumors. Therefore, we explored the differences in immune checkpoints (33) (**Supplementary Table S7**) and immune cell infiltration (**Supplementary Figure S4A**) between HCC and ICC and found that the immune microenvironment (65.9% of the immune checkpoints and 41.2% of the immune cells) between HCC and ICC is very different. More importantly, we are interested in the potential impact of alternative splicing on the immune microenvironment; thus, we performed a hierarchical consensus clustering analysis of HCC and ICC patients based on the hierarchical consensus clustering of DEAS events. Eventually, the HCC was divided into three groups (**Figure 4A** and **Supplementary Figures S4B**–**D**), and the ICC was divided into three groups (**Figure 4E** and **Supplementary Figures S4F**–**H**). We found that 45 immune checkpoints were significantly different between DEAS clusters in HCC, while only seven immune checkpoints in ICC (**Supplementary Tables S8**, **S9**). Interestingly, tryptophan 2,3-dioxygenase (TDO2) serves as a common immune metabolism checkpoint for HCC and ICC, which is consistent with the “cofactor metabolic process” previously identified as a common metabolic process between HCC and ICC. More importantly, PD-1 was significantly lower in cluster 2 than in cluster 1/3 (**Figure 4B**), while the expression of butyrophilin like 9 (BTNL9) in cluster 2 was significantly higher than that in cluster 1/3 in HCC (**Figure 4C**). In ICC, Poliovirus receptor (PVR), as another immunosuppression-related molecule, was significantly lower in cluster 2 than in clusters 1 and 3 (**Figure 4F**), while the expression of TNF receptor superfamily member 14 (TNFRSF14) in cluster 2 was significantly higher than that in cluster 3 (**Figure 4G**).

Next, we explored the differences in the infiltration of 34 types of immune cells in the DEAS clusters and found that 23 immune cells were considered significantly different between DEAS clusters in HCC (**Figure 4D**), while only one immune cell was considered significantly different between DEAS clusters in ICC (**Figure 4H**). In addition, the tumor microenvironment score and tumor stroma score based on DEAS clustering in HCC are meaningful, but not meaningful in ICC. Intriguingly, the components of “Th1 cells” show significant correlation with clusters both in HCC and ICC (**Supplementary Figures S4E, I**). In general, these results show that the immune microenvironment between HCC and ICC is very different, and more importantly, the correlation between AS and the immune microenvironment in HCC and ICC is extremely valuable for research.

### The Prognostic Value of DEAS Events in HCC and ICC

**Figure 5:**
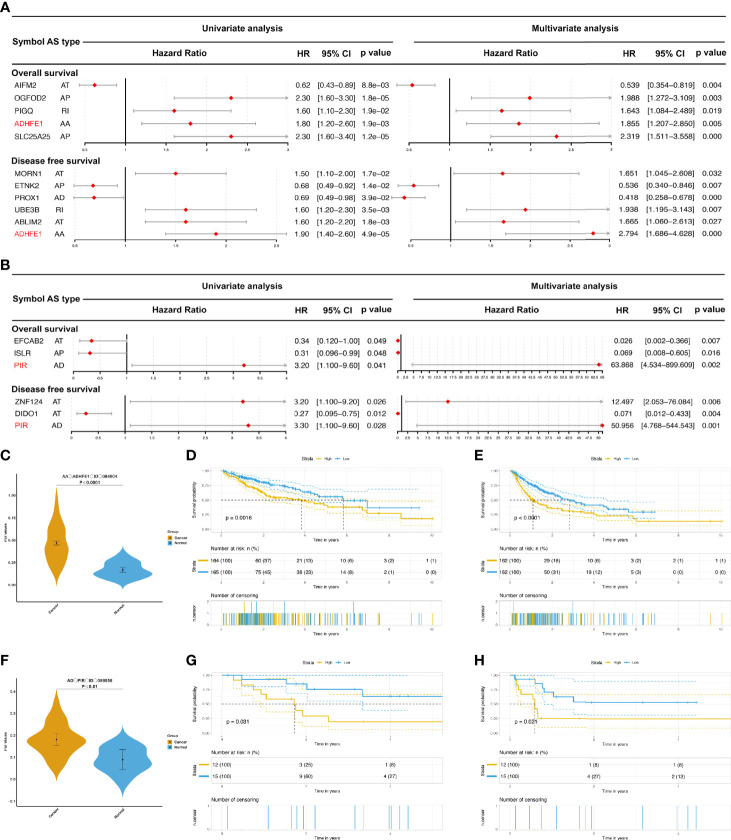
The prognostic value of DEAS events in HCC and ICC. **(A, B)** The forest map results of cox analysis of OS- and DFS-DEAS events in HCC **(A)** and ICC **(B)** were showed, and the common DEAS event in both OS and DFS was marked as red. **(C)** The difference PSI values of AA_ADHFE1_ID_084004 between normal tissues and HCC tissues. **(D, E)** Prognostic signatures based on AA_ADHFE1_ID_084004 in HCC for both OS **(D)** and DFS **(E)**. **(F)** The difference PSI values of AD_PIR_ID_008558 between normal tissues and HCC tissues. **(G, H)** Prognostic signatures based on AD_PIR_ID_008558 in HCC for both OS **(G)** and DFS **(H)**.

To further evaluate the importance of AS for HCC and ICC, we used univariate survival analysis (**Supplementary Tables S10**–**S11**) and LASSO regression analysis (**Supplementary Figure S5**) to analyze the impact of these DEAS on the survival and prognosis of HCC and ICC patients. And these DEAS events were selected in multivariate analysis. According to the results of multivariate survival analysis, five OS-DEAS events and six DFS-DEAS events were found as independent predictors of OS and DFS in patients with HCC (**Figure 5A**). In addition, in patients with ICC, three OS-DEAS events and three DFS-DEAS events were found as independent predictors (**Figure 5B**). Then we developed risk prediction formulas based on the above such DEAS events in HCC and ICC, respectively; the formulas are summarized in **Supplementary Table S12**.

### Nomogram Model Construction in HCC and ICC

**Figure 6:**
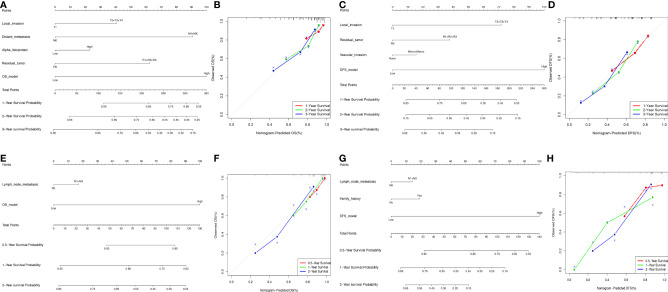
Nomogram model construction in HCC and ICC. **(A, B)** The development of nomogram to predict the 1-, 2-, and 3-year OS in HCC **(A)**, and the corresponding calibration **(B)**. **(C–D)** The development of nomogram to predict the 1-, 2-, and 3-year DFS in HCC **(C)**, and the corresponding calibration **(D)**. **(E, F)** The development of nomogram to predict the 0.5-, 1-, and 2-year OS in ICC **(E)**, and the corresponding calibration **(F)**. **(G, H)** The development of nomogram to predict the 0.5-, 1-, and 2-year OS in ICC **(G)**, and the corresponding calibration **(H)**.

The ideal predictive model should consider the importance of clinical data. Univariate survival analysis (**Tables 1**, **2**) and lasso regression (**Supplementary Figure S6**) were performed to identify suitable clinical predictors. Afterwards, four nomograms based on risk scores of above OS or DFS models and clinical variables were developed to predict the OS and DFS in patients with HCC (**Figures 6A, C**) or ICC (**Figures 6E, G**). In addition, the corresponding calibration curves of nomograms showed good agreement between the probability of prediction and observation in 1-, 2-, and 3-years OS and DFS in HCC (**Figures 6B, D**) and in the 0.5-, 1-, and 2-year OS and DFS in ICC (**Figures 6F, H**). The c-index of nomogram was 0.732 (95% CI: 0.671–0.793) in OS-HCC group, 0.683 (95% CI: 0.624–0.742) in DFS-HCC group, 0.762 (95% CI: 0.649–0.875) in OS-ICC group, and 0.771 (95% CI: 0.621–0.921) in DFS-ICC group (**Table 3**), and the ROC of four nomograms was also performed and is shown in **Supplementary Figure S7**, respectively. We also validate nomograms internally by randomly drawing 70% of the original cohort, and the results show that the c-index of nomograms was 0.8 in OS-HCC group, 0.77 in DFS-HCC group, 0.86 in OS-ICC group, and 0.82 in DFS-ICC group. These results show that four nomograms have good stability and distinguishing ability. Meanwhile, the c-index and AUC of all single variables included in the four nomograms were also identified, and the results showed that the c-index and AUC of almost all single predictors were lower than the nomogram. Only when predicting the 1-year DFS of ICC patients is the predictive power of the nomogram model slightly lower than that of the DFS-model (Nomo AUC: 0.849 *vs.* DFS-model AUC: 0.853).

**Table 1:** Univariate analyses of clinicopathological features for OS and DFS in HCC.

Characteristics	OS			DFS		
	HR	95% CI	P Value	HR	95% CI	P Value
Age (>60/≤60 years)	1.17	0.82–1.67	0.387	1	0.74–1.35	0.998
Sex (Male/Female)	1.25	0.87–1.8	0.225	1	0.72–1.37	0.977
BMI (≥25/<25)	0.72	0.5–1.04	0.081	0.86	0.64–1.17	0.339
Albumin (≥4/<4 g/dl))	0.84	0.55–1.28	0.415	0.87	0.62–1.22	0.417
Alpha_fetoprotein (>20/≤20 ng/ml)	1.75	1.1–2.77	***0.017***	1.35	0.95–1.91	0.094
Creatinine (≥1.1/<1.1 mg/dl)	0.76	0.48–1.2	0.235	0.7	0.48–1.01	0.055
Platelet (×10^9^/L)	1.39	0.9–2.16	0.142	1.3	0.93–1.82	0.130
Local_invasion (T2+T3+T4/T1)	2.33	1.6–3.4	***0.000***	2.39	1.75–3.26	***0.000***
Lymph_node_metastasis (N1+NX/N0)	1.62	1.11–2.35	***0.012***	1.26	0.92–1.74	0.155
Distant_metastasis (M1+MX/M0)	1.79	1.23–2.6	***0.002***	1.2	0.86–1.66	0.279
TNM_stage (Stage II+III+IV/Stage I)	2.31	1.56–3.45	***0.000***	2.32	1.68–3.19	***0.000***
Child_pugh_classification (B+C/A)	1.85	0.91–3.78	0.091	1.28	0.69–2.4	0.438
Adjacent_tissue_inflammation (Yes/No)	1.2	0.73–1.97	0.482	1.24	0.86–1.8	0.247
Family_history (Yes/No)	1.17	0.8–1.7	0.423	0.91	0.65–1.28	0.593
Race (White/Not_white)	1.25	0.86–1.81	0.242	1.33	0.98–1.8	0.069
Residual_tumor (R1+R2+RX/R0)	2.08	1.22–3.53	***0.007***	1.66	1.01–2.71	***0.044***
Vascular_invasion (Yes/No)	1.48	0.96–2.26	0.075	1.72	1.22–2.44	***0.002***
OS_model (High/low)	4.03	2.38–6.81	***0.000***			
DFS_model (High/low)				3	1.98–4.53	***0.000***

Italicized and bold, statistically significant.

OS, overall survival; DFS, disease-free survival; HCC, hepatocellular carcinoma; HR, hazard ratio; 95% CI, 95% confidence interval; BMI, body mass index; TNM, tumour_node_metastasis.

**Table 2:** Univariate analyses of clinicopathological features for OS and DFS in ICC.

Characteristics	OS			DFS		
	HR	95% CI	P Value	HR	95% CI	P Value
Age (>60/≤60 years)	0.95	0.34–2.63	0.916	0.75	0.3–1.89	0.539
Sex (Male/Female)	0.72	0.26–1.93	0.508	1.26	0.47–3.37	0.647
BMI (≥25/<25)	0.63	0.21–1.86	0.402	0.8	0.28–2.25	0.673
Albumin (≥4/<4 g/dl))	0.5	0.13–1.92	0.310	1.05	0.29–3.74	0.940
Creatinine (≥1.1/<1.1 mg/dl)	2.47	0.74–8.27	0.144	2.27	0.62–8.31	0.217
Platelet (×10^9^/L)	1.85	0.4–8.46	0.431	0.9	0.26–3.17	0.872
Local_invasion (T2+T3+T4/T1)	1.61	0.58–4.47	0.359	1.1	0.43–2.81	0.835
Lymph_node_metastasis (N1+NX/N0)	3.41	1.17–9.93	***0.025***	2.93	1.02–8.37	***0.045***
Distant_metastasis (M1+MX/M0)	1.29	0.36–4.57	0.697	1.28	0.42–3.93	0.662
TNM_stage (Stage II+III+IV/Stage I)	1.61	0.58–4.47	0.359	1.1	0.43–2.81	0.835
Child_pugh_classification (B+C/A)	2.13	0.25–18.42	0.491	1.02	0.13–8.11	0.983
Family_history (Yes/No)	0.51	0.18–1.41	0.191	0.36	0.14–0.97	***0.043***
Perineural_invasion (Yes/No)	3.41	0.84–13.83	0.087	1.43	0.46–4.44	0.535
Race (White/Not_white)	0.36	0.09–1.37	0.135	0.72	0.21–2.51	0.611
Residual_tumor (R1+R2+RX/R0)	1.92	0.61–6.05	0.266	1.03	0.3–3.58	0.961
OS_model (High/low)	7.41	1.94–28.22	***0.003***			
DFS_model (High/low)				16.28	3.46–76.57	***0.000***

Italicized and bold, statistically significant.

OS, overall survival; DFS, disease-free survival; ICC, intrahepatic cholangiocarcinoma; HR, hazard ratio; 95% CI, 95% confidence interval; BMI, body mass index; TNM, tumour_node_metastasis.

**Table 3:** C_index of the nomogram model variables in HCC and ICC.

Variables	C_index	95% CI
**HCC_OS**		
Nomogram	0.732	0.671–0.793
OS_model	0.679	0.636–0.722
Local_invasion	0.609	0.562–0.656
Alpha_fetoprotein	0.600	0.541–0.659
Residual_tumor	0.535	0.500–0.570
Distant_metastasis	0.534	0.489–0.579
**HCC_DFS**		
Nomogram	0.683	0.624–0.742
DFS_model	0.629	0.579–0.679
Local_invasion	0.626	0.588–0.664
Vascular_invasion	0.581	0.536–0.626
Residual_tumor	0.516	0.493–0.539
**ICC_OS**		
Nomogram	0.762	0.649–0.875
OS_model	0.754	0.652–0.856
Lymph_node_metastasis	0.629	0.507–0.751
**ICC_DFS**		
Nomogram	0.771	0.621–0.921
DFS_model	0.755	0.641–0.869
Family_history	0.606	0.482–0.730
Lymph_node_metastasis	0.601	0.498–0.704

HCC, hepatocellular carcinoma; ICC, intrahepatic cholangiocarcinoma; OS, overall survival; DFS, disease-free survival; 95% CI, 95% confidence interval.

### Verification of Vital DEAS Functions

As in the previous study, we found that AA_ADHFE1_ID_084004 was a common independent risk event for both OS and DFS in HCC (**Figure 5A**; ADHFE1, Alcohol Dehydrogenase Iron Containing 1). In addition, AD_PIR_ID_008558 was also a common independent risk factor for both OS and DFS in ICC (**Figure 5B**; PIR, Pirin). For intuitively showing the differences of AA_ADHFE1_ID_084004 and AD_PIR_ID_008558 between tumor and normal tissues, we performed graphs in scatter plot (**Figures 5C, F**). Based on the median value of PSI, patients were divided in high-risk group and low-risk group. The Kaplan-Meier curves showed that there are significant differences in two groups (**Figures 5D, E, G, H**). It is proved again that AA_ADHFE1_ID_084004 and AD_PIR_ID_008558 may play an important role in the development and recrudescence of HCC and ICC, respectively.

**Figure 7:**
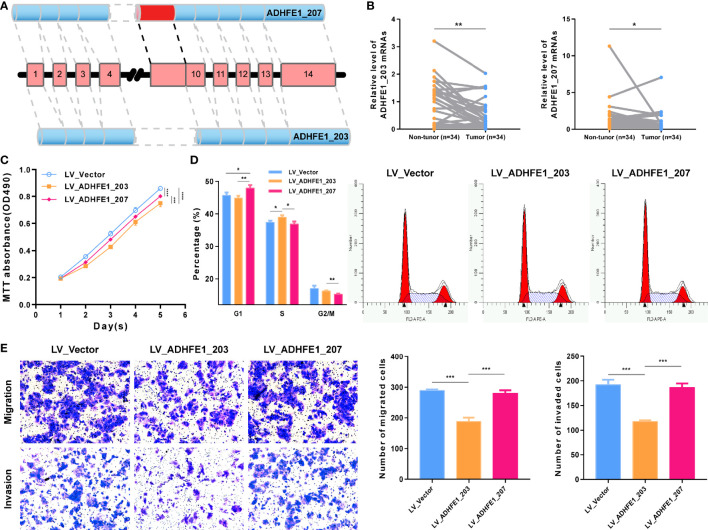
Functional experiment of ADHFE1 splicing variants in HCC cell. **(A)** The splicing pattern of ADHFE1_203 (ENST00000396623.8) and ADHFE1_207 (ENST00000424777.6). **(B)** The RNA expression of ADHFE1_203 and ADHFE1_207 in matched HCC and adjacent non-tumor frozen tissues. **(C–E)** MTT assay **(C)**, Cell Cycle analysis **(D)**, and Transwell assays **(E)**. *P < 0.05, **P < 0.01, ***P < 0.001, ****P < 0.0001.

To further verify that AA_ADHFE1_ID_084004 is very important to the development and recrudescence of HCC, we verify the function of two transcripts related to DEAS by experiments. The top left plot displays the splicing pattern of ADHFE1_203 and ADHFE1_207 [ADHFE1, through Alternate acceptor site (AA), an alternative splicing type, produced two transcripts] (**Figure 7A**), and the top right plot shows that the expression of ADHFE1_203 and ADHFE1_207 in HCC is significantly lower than that in adjacent non-tumor frozen tissues (**Figure 7B**). More importantly, MTT assay suggested that the overexpression of ADHFE1_203 and ADHFE1_207 inhibited the proliferation of HCC cells (**Supplementary Figure S8A** and **Figure 7C**), and Cell Cycle analysis suggested that the overexpression of ADHFE1_203 inhibited S phase, while the overexpression of ADHFE1_207 inhibited G1 phase (**Figure 7D**). In addition, we observed that the overexpression of ADHFE1_203 significantly inhibited migration and invasion in Huh7 cell line (**Figure 7E**). Intriguingly, MTT and transwell assays showed that compared with the overexpression of ADHFE1_207, the overexpression of ADHFE1_203 had a stronger ability to inhibit proliferation, invasion, and migration. These results directly indicated that DEAS events were important biological processes and had a potential clinical value.

### Correlation Analyses Between SFs and OS-/DFS-DEAS Events in HCC and ICC

**Figure 8:**
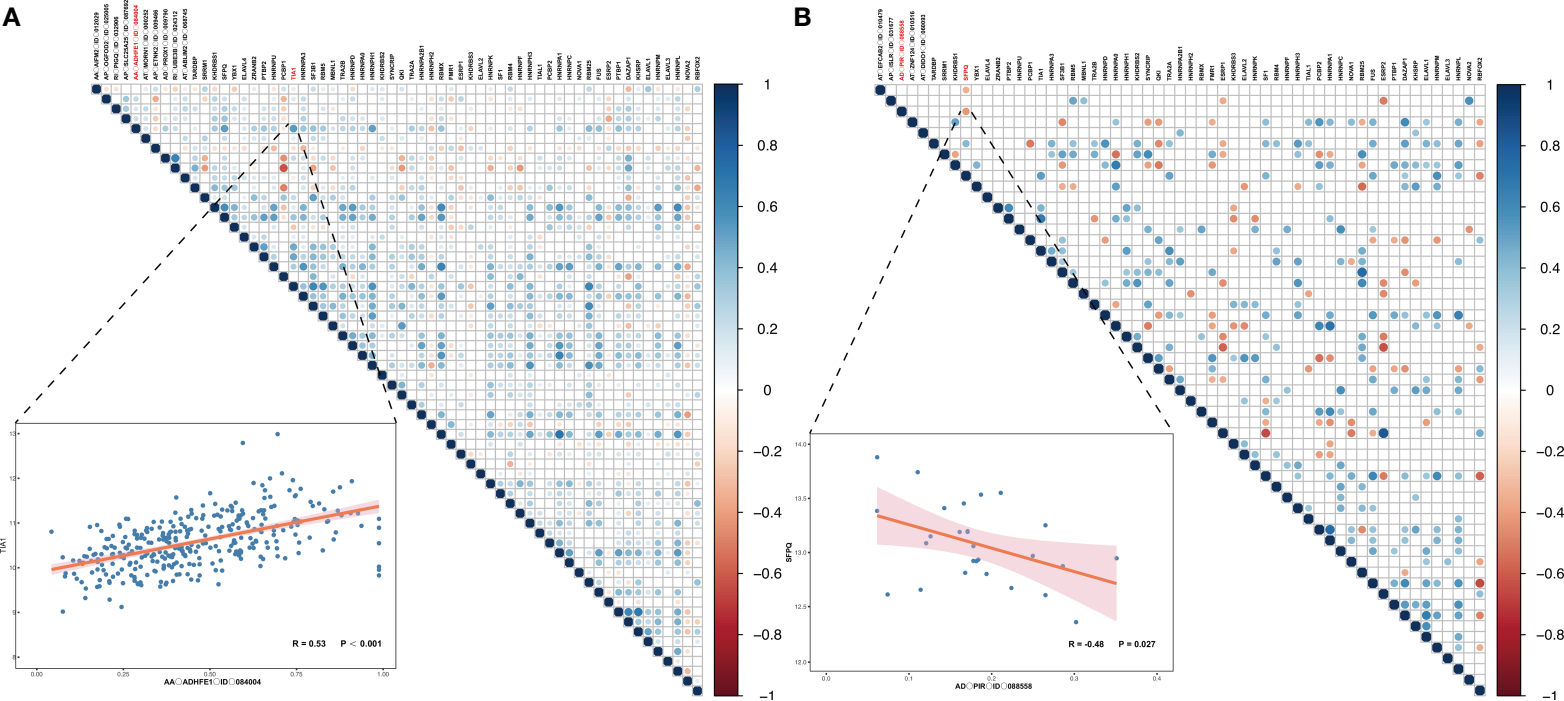
Correlation analyses between SFs and OS-/DFS-DEAS events in HCC and ICC. **(A, B)** The correlation between SFs and OS- and DFS-DEAS events in HCC **(A)** and ICC **(B)**. The right figure displays the significance and the correlation coefficient between the expression of SFs and the PSI values of OS-/DFS-DEAS events. If there is a circle, P <0.05, and the size of the circle represents the size of P value. The color of the circle represents the correlation coefficient. The left figure demonstrates the correlation between the PSI values of representative DEAS events and expression of SF.

So, what are the reasons for the emergence of important DEAS such as AA_ADHFE1_ID_084004 and AD_PIR_ID_008558? As we all know, SFs are important factors to regulate the DEAS events. Hence, we further studied which SF can regulate the production of OS- and DFS-DEAS events. Thus, correlation analyses between expression levels of the PSI values of these DEAS events and 71 SFs (**Supplementary Table S13**) were conducted to explore the candidate regulation network in the HCC and ICC (34). As shown in **Figures 8A, B**, we can find that most of the SFs positively related with these DEAS events [67.11% (204/304) in HCC and 61.54% (16/26) in ICC]. In addition, we can also find that most single SF was correlated with more than one DEAS events, and the number of AS events correlated with some SFs even reach nine (DAZAP1 and HNRNPL in HCC). Further, we identified the SFs that significantly correlated with common DEAS events determined above (AA_ADHFE1_ID_084004 and AD_PIR_ID_008558). And we found that a lot of SFs correlated with AA_ADHFE1_ID_084004, but the T-cell intracellular antigen 1 (TIA1) is the most significantly correlated SFs (r=0.532, p<0.001). However, only SF Proline and Glutamine Rich (SFPQ) was identified correlated with AD_PIR_ID_008558 (r=−0.424, p=0.027).

More importantly, to better understand the specific splicing mechanism of SFs, we further explored the protein crystal structure of TIA1 and SFPQ. Analysis of TIA1 sequence and structures indicates it contains three RNA recognition motif (RRM), and each RRM structure consists of four antiparallel strands and two helices arranged in an alpha/beta sandwich, with beta sheet interacting with RNA molecules. **Supplementary Figure S8B** highlights interactions observed in the published crystal structures. Analysis of SFPQ sequences reveal it also contains two RRMs. Though available crystal structures of SFPQ do not contain RNA, structure-based alignment of SFPQ (PDB code 6NCQ) with TIA1 (PDB code 5O3J) indicates that SFPQ is capable of recognizing RNA (**Supplementary Figure S8C**).

## Discussion

Recently, some studies reported that AS event significantly related with apoptosis, angiogenesis, and immunology (35, 36). And the correlation between tumor and AS event was gradually discovered, which was considered to play an important role in tumorigenesis, invasion, and drug resistance of cancer (37–41). In the present studies, 384 and 749 DEAS events were confirmed in HCC and ICC, respectively. Moreover, the relationship between DEAS events and biological process or immune features has also been initially recognized. Most notably, we found that the DEAS events and its molecular mechanism in two subtypes of PCL have obvious differences. Subsequently, we subdivided and verified the specific DEAS events and developed four nomograms to predict the prognosis. And we also found that the relationship of SFs and DEAS events provided a potential regulatory mechanism for the abnormal changes in HCC and ICC and further showed some protein crystal structure. Our joint and integrated investigation focused on the DEAS events of HCC and ICC, which has an important impetus for understanding the pathogenesis, predicting the progression, and further treatment such disease.

As is known to us, liver and bile duct originate from the same hepatic diverticulum of endoderm. However, ICC is more malignant than HCC and has a poor prognosis (42). For HCC patients undergoing therapeutic surgery, the 5-year overall survival rate is about 50–70% (43, 44), which is much higher than that of ICC patients undergoing therapeutic surgery (20–40%) (45, 46). Therefore, it is particularly important to explore the mechanism to promote the occurrence and development of HCC and ICC. Compared with the previous studies, the most important highlight in our study is that we jointly analyzed the differences in two common histological type of liver cancer in the level of DEAS events. For example, although we found HCC and ICC are partly common in DEAS events or DEG, more of them are different between HCC and ICC. Besides, the potential functional analyses of the parent genes of DEAS found that they may play significant roles in tumorigenesis and metabolic process. Intriguingly, “cofactor metabolic process” and “ lipid biosynthetic process” were identified as the common pathway of HCC and ICC in GO analysis. And the “Ferroptosis” was identified as the common pathway by KEGG analysis, which has been confirmed was related with head and neck cancer and lung cancer (47, 48). These common targets are expected to be new potential therapeutic targets. But it should point that the differences between such two common histological types of PCL still dominate in major biological process.

The immune microenvironment of HCC and ICC is quite different (49), and the role of alternative splicing in the formation of the immune microenvironment is worth exploring. After clustering by DEAS events, it is found that HCC and ICC have a common immune metabolic checkpoint TDO2 (50), which is consistent with the “cofactor metabolic process” that we have enriched in functions. More importantly, PD-1 and PVR have been reported in previous studies as an immunosuppressive factor (51, 52), while BTNL9 and TNFRSF14 have been reported as an immune activating factor (53, 54). This is consistent with the results of our cluster analysis based on DEAS. In HCC, the PD-1 expression of cluster 2 was significantly lower than that of clusters 1 and 3, while the expression of BTNL9 of cluster 2 was significantly higher than that of clusters 1 and 3. In ICC, the PVR expression of cluster 2 was significantly lower than that of clusters 1 and 3, while the expression of TNFRSF14 of cluster 2 was significantly higher than that of cluster 3. In addition, we also explored the infiltration of 34 immune cells in HCC and ICC and found that only the components of “Th1 cells” showed significant correlation with clusters both in HCC and ICC. Previous studies have shown that Th1 cells play a very important role in the occurrence and development of HCC and ICC (55, 56), which is consistent with our research. This shows that cluster analysis based on DEAS can accurately reflect the differences in the immune microenvironment of HCC and ICC, which provides a theoretical basis for the development of HCC and ICC immunotherapies to prolong patient survival. Although HCC and ICC are hepatogenic malignancies, there are great differences in the prognosis between them. The poor prognosis of ICC may be due to the lack of immune infiltration and the lack of specific immunotherapeutic targets. Therefore, HCC and ICC should be accurately distinguished. When treating common targets, specific target therapy should be combined to achieve accurate treatment and further improve the prognosis of patients.

In the previous studies, ADHFE1 has been shown to be closely related with the tumorigenesis and progression of many cancers (57, 58). In the present study, AA_ADHFE1_ID_084004 was identified as an independent risk AS event for OS and demonstrated the same trend in DFS. Intriguingly, although ADHFE1 was downregulated, Alternate acceptor site (AA) event in ADHFE1 was upregulated in HCC. This is consistent with the results of our cell function experiment. We found that ADHFE1_203 has a stronger anticancer effect than ADHFE1_207. It may be that the occurrence of AA events represents an increase in the proportion of ADHFE1_207s, and the overall anticancer effect is weakened, so it indicates a poor prognosis. In addition, another AS event that was identified as an independent prognostic risk factor for both OS and DFS in ICC patients is AD_PIR_ID_008558. Its parent gene has been confirmed to be associated with tumorigenesis (59). Although the difference of the PIR expression was not statistically significant between tumor and normal tissues, AD event in PIR was significantly upregulated in ICC. This suggests that DEAS events may play a more important role in tumor progression than its parent gene, which is a research orientation in the future.

Due to the poor prognosis of liver cancer, it is important to develop a predictive tool to predict the prognosis of patients. Up to now, the predictive tool based on clinicopathological, laboratory tests, radiology results, methylation markers, or miRNA have been established for liver cancer patients (60–66). However, the nomogram based on the DEAS events to predict the prognosis of liver cancer is lacking, no matter HCC or ICC. In fact, the nomogram based on AS events and clinicopathological has been developed in breast cancer and showed good performance of discrimination and clinical usefulness (67). In the present study, four nomograms were developed based on the clinical variables and risk scores, which were calculated by the OS-DEAS events or DFS-DEAS events. C-index and AUC of nomogram indicated that the discrimination of all nomograms is well. More importantly, all of C-index and almost of AUCs in nomogram were higher than any single predictors in nomograms. These data demonstrate that the nomogram combined with DEAS events has high potential prognostic value in HCC patients.

It is worth noting that a single SF usually regulates more than one DEAS event, and the different SFs may even show an opposite regulatory effect on the same DEAS events. This shows that the regulation of SF is a complex network (20). In our research, TIA1 was identified as ADHFE1-correlated SF. Literature reports suggested TIA1 RRM2 is primarily involved in recognizing U-rich sequences while RRM3 preferentially interacts with C-rich sequences (68). We highlight interactions observed in the published crystal structures. Moreover, as is validated in previous research, TIA1 is an important tumor suppressor involved in many aspects of carcinogenesis and cancer development. It can regulate tumor cell proliferation, migration in gastric cancer, colorectal cancer, and esophageal squamous cell carcinoma (69–71). Besides, SFPQ was PIR-correlated SF, and the structure-based alignment of SFPQ with TIA1 indicates that SFPQ is capable of recognizing RNA. In addition, the gene of SFPQ was also identified as tumor-related gene. Many molecules can facilitate proliferation, migration, and invasion of cancer cells by targeting SFPQ (72, 73). Therefore, a better understanding of the specific splicing mechanism of SF may allow a novel idea for improving patient survival.

In summary, the potential mechanisms and immune functions of DEAS events in HCC and ICC were identified in the present study. Despite some similarities between HCC and ICC were found in the AS level, it should be noticed the difference between them accounts for a greater part. In addition, the results of our study highlight the prognostic significance of DEAS event in HCC and ICC, and the predictive models developed have shown the great clinical utilization value. These results might provide new insight in HCC and ICC prevention and treatment.

## Data Availability Statement

The original contributions presented in the study are included in the article/**Supplementary Material**. Further inquiries can be directed to the corresponding author.

## Author Contributions

BH and CL designed the project. DL, DZ, MZ, CH, HL and SZ collected and assembled the data. DL, DZ, MZ, XC, LH, BL and CJ analyzed and interpreted the data. DL, DZ, and MZ drafted the manuscript. CL and BH provided the financial support. All authors contributed to the article and approved the submitted version.

## Funding

This work was supported by grants from the Shandong Provincial Natural Science Foundation of China (grant number ZR2020MH217) and the Key Research and Development Plan of Shandong Province (grant number 2018GSF118233).

## Conflict of Interest

The authors declare that the research was conducted in the absence of any commercial or financial relationships that could be construed as a potential conflict of interest.

## Publisher’s Note

All claims expressed in this article are solely those of the authors and do not necessarily represent those of their affiliated organizations, or those of the publisher, the editors and the reviewers. Any product that may be evaluated in this article, or claim that may be made by its manufacturer, is not guaranteed or endorsed by the publisher.

## References

[1] Fitzmaurice C Abate D Abbasi N Abbastabar H Abd-Allah F Abdel-Rahman O. Global, Regional, and National Cancer Incidence, Mortality, Years of Life Lost, Years Lived With Disability, and Disability-Adjusted Life-Years for 29 Cancer Groups, 1990 to 2017: A Systematic Analysis for the Global Burden of Disease Study. JAMA Oncol (2019) 4(11):1553–68. doi: 10.1001/jamaoncol.2019.2996 PMC6248091 29860482

[2] Allemani C Matsuda T Di Carlo V Harewood R Matz M Nikšic M. Global Surveillance of Trends in Cancer Survival 2000-14 (CONCORD-3): Analysis of Individual Records for 37 513 025 Patients Diagnosed With One of 18 Cancers From 322 Population-Based Registries in 71 Countries. Lancet (2018) 391(10125):1023–75. doi: 10.1016/S0140-6736(17)33326-3 PMC5879496 29395269

[3] Jemal A Bray F Center MM Ferlay J Ward E Forman DJ. Global Cancer Statistics. CCJC (2011) 61(2):69–90. doi: 10.3322/caac.20107 21296855

[4] Cardinale V Bragazzi MC Carpino G Torrice A Alvaro D. Cholangiocarcinoma: Increasing Burden of Classifications. Hepatobiliary Surg Nutr (2013) 2(5):272–80. doi: 10.3978/j.issn.2304-3881.2013.10.02 PMC3924690 24570958

[5] Hoadley KA Yau C Hinoue T Wolf DM Lazar AJ Drill E. Cell-Of-Origin Patterns Dominate the Molecular Classification of 10,000 Tumors From 33 Types of Cancer. Cell (2018) 173(2):291–304.e6. doi: 10.1016/j.cell.2018.03.022 29625048 PMC5957518

[6] Wolk K Witte E Witte K Warszawska K Sabat R. Biology of Interleukin-22. Semin Immunopathol (2010) 32(1):17–31. doi: 10.1007/s00281-009-0188-x 20127093

[7] Yang X Coulombe-Huntington J Kang S Sheynkman GM Hao T Richardson A. Widespread Expansion of Protein Interaction Capabilities by Alternative Splicing. Cell (2016) 164(4):805–17. doi: 10.1016/j.cell.2016.01.029 PMC4882190 26871637

[8] Porazinski S Ladomery MJG. Alternative Splicing in the Hippo Pathway—Implications for Disease and Potential Therapeutic Targets. Genes (2018) 9(3):161. doi: 10.3390/genes9030161 PMC5867882 29534050

[9] Wu H-Y Wei Y Liu L-M Chen Z-B Hu Q-P Pan S-L. Construction of a Model to Predict the Prognosis of Patients With Cholangiocarcinoma Using Alternative Splicing Events. Oncol Lett (2019) 18(5):4677–90. doi: 10.3892/ol.2019.10838 PMC6781777 31611977

[10] Dong S Lu L-J. An Alternative Splicing Signature Model for Predicting Hepatocellular Carcinoma-Specific Survival. J Gastrointest Oncol (2020) 11(5):1054–64. doi: 10.21037/jgo-20-377 PMC7657838 33209497

[11] Xiong Y Yang G Wang K Riaz M Xu J Lv Z. Genome-Wide Transcriptional Analysis Reveals Alternative Splicing Event Profiles in Hepatocellular Carcinoma and Their Prognostic Significance. Front Genet (2020) 11:879. doi: 10.3389/fgene.2020.00879 32849842 PMC7432180

[12] Yao J Caballero OL Huang Y Lin C Rimoldi D Behren A. Altered Expression and Splicing of ESRP1 in Malignant Melanoma Correlates With Epithelial-Mesenchymal Status and Tumor-Associated Immune Cytolytic Activity. Cancer Immunol Res (2016) 4(6):552–61. doi: 10.1158/2326-6066.cir-15-0255 27045022

[13] Kim EK Yoon SO Jung WY Lee H Kang Y Jang YJ. Implications of NOVA1 Suppression Within the Microenvironment of Gastric Cancer: Association With Immune Cell Dysregulation. Gastric Cancer (2017) 20(3):438–47. doi: 10.1007/s10120-016-0623-3 27318497

[14] Padthaisong S Thanee M Namwat N Phetcharaburanin J Klanrit P Khuntikeo N. Overexpression of a Panel of Cancer Stem Cell Markers Enhances the Predictive Capability of the Progression and Recurrence in the Early Stage Cholangiocarcinoma. J Transl Med (2020) 18(1):64. doi: 10.1186/s12967-020-02243-w 32039729 PMC7008521

[15] Taylor JS Rajan SS Zhang N Meyer LA Ramondetta LM Bodurka DC. End-Of-Life Racial and Ethnic Disparities Among Patients With Ovarian Cancer. J Clin Oncol (2017) 35(16):1829–35. doi: 10.1200/JCO.2016.70.2894 PMC5455594 28388292

[16] Zaghdoudi S Decaup E Belhabib I Samain R Cassant-Sourdy S Rochotte J. FAK Activity in Cancer-Associated Fibroblasts Is a Prognostic Marker and a Druggable Key Metastatic Player in Pancreatic Cancer. EMBO Mol Med (2020) 12(11):e12010. doi: 10.15252/emmm.202012010 33025708 PMC7645544

[17] Wang Z Jensen MA Zenklusen JC. A Practical Guide to The Cancer Genome Atlas (TCGA). Methods Mol Biol (2016) 1418:111–41. doi: 10.1007/978-1-4939-3578-9_6 27008012

[18] Love MI Huber W Anders SJGB. Moderated Estimation of Fold Change and Dispersion for RNA-Seq Data With Deseq2. Genome Biol (2014) 15(12):550. doi: 10.1186/s13059-014-0550-8 25516281 PMC4302049

[19] Ryan M Wong WC Brown R Akbani R Su X Broom B. TCGASpliceSeq a Compendium of Alternative mRNA Splicing in Cancer. Nucleic Acids Res (2016) 44(Database issue):D1018–22. doi: 10.1093/nar/gkv1288 PMC4702910 26602693

[20] Xiong Y Deng Y Wang K Zhou H Zheng X Si L. Profiles of Alternative Splicing in Colorectal Cancer and Their Clinical Significance: A Study Based on Large-Scale Sequencing Data. EBioMedicine (2018) 36:183–95. doi: 10.1016/j.ebiom.2018.09.021 PMC6197784 30243491

[21] Conway JR Lex A Gehlenborg N. UpSetR: An R Package for the Visualization of Intersecting Sets and Their Properties. Bioinformatics (Oxford England) (2017) 33(18):2938–40. doi: 10.1093/bioinformatics/btx364 PMC5870712 28645171

[22] Krzywinski M Schein J Birol I Connors J Gascoyne R Horsman D. Circos: An Information Aesthetic for Comparative Genomics. Genome Res (2009) 19(9):1639–45. doi: 10.1101/gr.092759.109 PMC2752132 19541911

[23] Szklarczyk D Morris JH Cook H Kuhn M Wyder S Simonovic M. The STRING Database in 2017: Quality-Controlled Protein-Protein Association Networks, Made Broadly Accessible. Nucleic Acids Res (2017) 45(D1):D362–d8. doi: 10.1093/nar/gkw937 PMC5210637 27924014

[24] Su G Morris JH Demchak B Bader GD. Biological Network Exploration With Cytoscape 3. Curr Protoc Bioinformatics (2014) 47:8.13.1–24. doi: 10.1002/0471250953.bi0813s47 PMC4174321 25199793

[25] Wilkerson MD Hayes DN. ConsensusClusterPlus: A Class Discovery Tool With Confidence Assessments and Item Tracking. Bioinformatics (Oxford England) (2010) 26(12):1572–3. doi: 10.1093/bioinformatics/btq170 PMC2881355 20427518

[26] Yoshihara K Shahmoradgoli M Martinez E Vegesna R Kim H Torres-Garcia W. Inferring Tumour Purity and Stromal and Immune Cell Admixture From Expression Data. Nat Commun (2013) 4:2612. doi: 10.1038/ncomms3612 24113773 PMC3826632

[27] Ye L Zhang T Kang Z Guo G Sun Y Lin K. Tumor-Infiltrating Immune Cells Act as a Marker for Prognosis in Colorectal Cancer. Front Immunol (2019) 10:2368. doi: 10.3389/fimmu.2019.02368 31681276 PMC6811516

[28] Tibshirani RJ. Regression Shrinkage and Selection via the Lasso. JotRSSSB (1996) 58(1):267–88. doi: 10.1111/j.2517-6161.1996.tb02080.x

[29] Harrell FE. R Software. Regression Modeling Strategies: with Applications to Linear Models, Logistic and Ordinal Regression, and Survival Analysis, 2nd Edition. Springer International Publishing Ag (2015). p.127–42. doi: 10.1007/978-3-319-19425-7

[30] Vickers AJ Elkin EB. Decision Curve Analysis: A Novel Method for Evaluating Prediction Models. JMDM (2006) 26(6):565–74. doi: 10.1177/0272989X06295361 PMC2577036 17099194

[31] Heagerty PJ Lumley T Pepe MS. Time-Dependent ROC Curves for Censored Survival Data and a Diagnostic Marker. JB (2000) 56(2):337–44. doi: 10.1111/j.0006-341X.2000.00337.x 10877287

[32] Giulietti M Piva F D’Antonio M Meo PDOD Paoletti D Castrignanò T. SpliceAid-F: A Database of Human Splicing Factors and Their RNA-Binding Sites. Nucleic Acids Res (2013) 41(Database issue):D125. doi: 10.1093/nar/gks997 23118479 PMC3531144

[33] Hu FF Liu CJ Liu LL Zhang Q Guo AY. Expression Profile of Immune Checkpoint Genes and Their Roles in Predicting Immunotherapy Response. Brief Bioinform (2020) 22(3). doi: 10.1093/bib/bbaa176 32814346

[34] Piva F Giulietti M Burini AB Principato G. SpliceAid 2: A Database of Human Splicing Factors Expression Data and RNA Target Motifs. Hum Mutat (2012) 33(1):81–5. doi: 10.1002/humu.21609 21922594

[35] Stevens M Oltean S. Modulation of the Apoptosis Gene Bcl-X Function Through Alternative Splicing. Front Genet (2019) 10:804. doi: 10.3389/fgene.2019.00804 31552099 PMC6743414

[36] Bowler E Oltean S. Alternative Splicing in Angiogenesis. Int J Mol Sci (2019) 20(9). doi: 10.3390/ijms20092067 PMC6540211 31027366

[37] Olender J Lee NH. Role of Alternative Splicing in Prostate Cancer Aggressiveness and Drug Resistance in African Americans. Med LNJAie Biol (2019) 1164(undefined):119–39. doi: 10.1007/978-3-030-22254-3_10 PMC6777849 31576545

[38] Amirkhah R Naderi-Meshkin H Shah JS Dunne PD Schmitz U. The Intricate Interplay Between Epigenetic Events, Alternative Splicing and Noncoding RNA Deregulation in Colorectal Cancer. Cells (2019) 8(8). doi: 10.3390/cells8080929 PMC6721676 31430887

[39] Paschalis A Sharp A Welti JC Neeb A Raj GV Luo J. Alternative Splicing in Prostate Cancer. Nat Rev Clin Oncol (2018) 15(11):663–75. doi: 10.1038/s41571-018-0085-0 30135575

[40] Siegfried Z Karni R. The Role of Alternative Splicing in Cancer Drug Resistance. Curr Opin Genet Dev (2018) 48:16–21. doi: 10.1016/j.gde.2017.10.001 29080552

[41] Marozin S Altomonte J Stadler F Thasler WE Schmid RM Ebert O. Inhibition of the IFN-Beta Response in Hepatocellular Carcinoma by Alternative Spliced Isoform of IFN Regulatory Factor-3. Mol Ther (2008) 16(11):1789–97. doi: 10.1038/mt.2008.201 18781139

[42] Zhou XD Tang ZY Fan J Zhou J Wu ZQ Qin LX. Intrahepatic Cholangiocarcinoma: Report of 272 Patients Compared With 5,829 Patients With Hepatocellular Carcinoma. J Cancer Res Clin Oncol (2009) 135(8):1073–80. doi: 10.1007/s00432-009-0547-y PMC12160141 19294418

[43] Yang JD Hainaut P Gores GJ Amadou A Plymoth A Roberts LR. A Global View of Hepatocellular Carcinoma: Trends, Risk, Prevention and Management. Nat Rev Gastroenterol Hepatol (2019) 16(10):589–604. doi: 10.1038/s41575-019-0186-y 31439937 PMC6813818

[44] Marrero JA Kulik LM Sirlin CB Zhu AX Finn RS Abecassis MM. Diagnosis, Staging, and Management of Hepatocellular Carcinoma: 2018 Practice Guidance by the American Association for the Study of Liver Diseases. Hepatology (2018) 68(2):723–50. doi: 10.1002/hep.29913 29624699

[45] Mavros MN Economopoulos KP Alexiou VG Pawlik TM. Treatment and Prognosis for Patients With Intrahepatic Cholangiocarcinoma: Systematic Review and Meta-Analysis. JAMA Surg (2014) 149(6):565–74. doi: 10.1001/jamasurg.2013.5137 24718873

[46] Bridgewater J Galle PR Khan SA Llovet JM Park JW Patel T. Guidelines for the Diagnosis and Management of Intrahepatic Cholangiocarcinoma. J Hepatol (2014) 60(6):1268–89. doi: 10.1016/j.jhep.2014.01.021 24681130

[47] Lin R Zhang Z Chen L Zhou Y Zou P Feng C. Dihydroartemisinin (DHA) Induces Ferroptosis and Causes Cell Cycle Arrest in Head and Neck Carcinoma Cells. Cancer Lett (2016) 381(1):165–75. doi: 10.1016/j.canlet.2016.07.033 27477901

[48] Kuang Y Wang Q. Iron and Lung Cancer. Cancer Lett (2019) 464:56–61. doi: 10.1016/j.canlet.2019.08.007 31437477

[49] Martin-Sierra C Martins R Laranjeira P Coucelo M Abrantes AM Oliveira RC. Functional and Phenotypic Characterization of Tumor-Infiltrating Leukocyte Subsets and Their Contribution to the Pathogenesis of Hepatocellular Carcinoma and Cholangiocarcinoma. Transl Oncol (2019) 12(11):1468–79. doi: 10.1016/j.tranon.2019.07.019 PMC6712279 31425839

[50] Sadik A Somarribas Patterson LF Ozturk S Mohapatra SR Panitz V Secker PF. IL4I1 Is a Metabolic Immune Checkpoint That Activates the AHR and Promotes Tumor Progression. Cell (2020) 182(5):1252–70.e34. doi: 10.1016/j.cell.2020.07.038 32818467

[51] Cancer Genome Atlas Research Network. Electronic Address Wbe, Cancer Genome Atlas Research N. Comprehensive and Integrative Genomic Characterization of Hepatocellular Carcinoma. Cell (2017) 169(7):1327–41.e23. doi: 10.1016/j.cell.2017.05.046 28622513 PMC5680778

[52] O'Donnell JS Madore J Li XY Smyth MJ. Tumor Intrinsic and Extrinsic Immune Functions of CD155. Semin Cancer Biol (2020) 65:189–96. doi: 10.1016/j.semcancer.2019.11.013 31883911

[53] Fang J Chen F Liu D Gu F Chen Z Wang Y. Prognostic Value of Immune Checkpoint Molecules in Breast Cancer. Biosci Rep (2020) 40(7). doi: 10.1042/BSR20201054 PMC7340863 32602545

[54] Ren S Tian Q Amar N Yu H Rivard CJ Caldwell C. The Immune Checkpoint, HVEM may Contribute to Immune Escape in Non-Small Cell Lung Cancer Lacking PD-L1 Expression. Lung Cancer (2018) 125:115–20. doi: 10.1016/j.lungcan.2018.09.004 30429008

[55] Behboudi S Alisa A Boswell S Anastassiou J Pathan AA Williams R. Expansion of Anti-AFP Th1 and Tc1 Responses in Hepatocellular Carcinoma Occur in Different Stages of Disease. Br J Cancer (2010) 102(4):748–53. doi: 10.1038/sj.bjc.6605526 PMC2837570 20087354

[56] Sadeghlar F Vogt A Mohr RU Mahn R van Beekum K Kornek M. Induction of Cytotoxic Effector Cells Towards Cholangiocellular, Pancreatic, and Colorectal Tumor Cells by Activation of the Immune Checkpoint CD40/CD40L on Dendritic Cells. Cancer Immunol Immunother (2020) 70(5):1451–64. doi: 10.1007/s00262-020-02746-x PMC8053193 33180184

[57] Hu YH Ma S Zhang XN Zhang ZY Zhu HF Ji YH. Hypermethylation Of ADHFE1 Promotes The Proliferation Of Colorectal Cancer Cell Via Modulating Cell Cycle Progression. Onco Targets Ther (2019) 12:8105–15. doi: 10.2147/ott.S223423 PMC6782030 31632063

[58] Mishra P Tang W Ambs S. ADHFE1 Is a MYC-Linked Oncogene That Induces Metabolic Reprogramming and Cellular De-Differentiation in Breast Cancer. Mol Cell Oncol (2018) 5(3):e1432260. doi: 10.1080/23723556.2018.1432260 30250890 PMC6150044

[59] Suleman M Chen A Ma H Wen S Zhao W Lin D. PIR Promotes Tumorigenesis of Breast Cancer by Upregulating Cell Cycle Activator E2F1. Cell Cycle (Georgetown, Tex) (2019) 18(21):2914–27. doi: 10.1080/15384101.2019.1662259 PMC6791709 31500513

[60] Berardi G Morise Z Sposito C Igarashi K Panetta V Simonelli I. Development of a Nomogram to Predict Outcome After Liver Resection for Hepatocellular Carcinoma in Child-Pugh B Cirrhosis. J Hepatol (2019) 72(1):75–84. doi: 10.1016/j.jhep.2019.08.032 31499131

[61] Lee S Kang TW Song KD Lee MW Rhim H Lim HK. Effect of Microvascular Invasion Risk on Early Recurrence of Hepatocellular Carcinoma After Surgery and Radiofrequency Ablation. Ann Surg (2019) 273(3):564–71. doi: 10.1097/sla.0000000000003268 31058694

[62] Xu X Zhang HL Liu QP Sun SW Zhang J Zhu FP. Radiomic Analysis of Contrast-Enhanced CT Predicts Microvascular Invasion and Outcome in Hepatocellular Carcinoma. J Hepatol (2019) 70(6):1133–44. doi: 10.1016/j.jhep.2019.02.023 30876945

[63] Long J Zhang L Wan X Lin J Bai Y Xu W. A Four-Gene-Based Prognostic Model Predicts Overall Survival in Patients With Hepatocellular Carcinoma. J Cell Mol Med (2018) 22(12):5928–38. doi: 10.1111/jcmm.13863 PMC6237588 30247807

[64] Lu CY Chen SY Peng HL Kan PY Chang WC Yen CJ. Cell-Free Methylation Markers With Diagnostic and Prognostic Potential in Hepatocellular Carcinoma. Oncotarget (2017) 8(4):6406–18. doi: 10.18632/oncotarget.14115 PMC5351641 28031532

[65] Zhang L Xiang ZL Zeng ZC Fan J Tang ZY Zhao XJ. A microRNA-Based Prediction Model for Lymph Node Metastasis in Hepatocellular Carcinoma. Oncotarget (2016) 7(3):3587–98. doi: 10.18632/oncotarget.6534 PMC4823129 26657296

[66] El-Serag EHB El-Serag F Davila JA Kramer J Richardson P. Gastroenterology RPJ. A New Laboratory-Based Algorithm to Predict Development of Hepatocellular Carcinoma in Patients With Hepatitis C and Cirrhosis. Gastroenterology (2014) 146(5):1249–55.e1.. doi: 10.1053/j.gastro.2014.01.045 PMC3992177 24462733

[67] Zhang D Duan Y Cun J Yang Q. Identification of Prognostic Alternative Splicing Signature in Breast Carcinoma. Front Genet (2019) 10:278. doi: 10.3389/fgene.2019.00278 30984247 PMC6448481

[68] Waris S García-Mauriño SM Sivakumaran A Beckham SA Loughlin FE Gorospe M. TIA-1 RRM23 Binding and Recognition of Target Oligonucleotides. Nucleic Acids Res (2017) 45(8):4944–57. doi: 10.1093/nar/gkx102 PMC5416816 28184449

[69] Yang X Wang M Lin B Yao D Li J Tang X. miR-487a Promotes Progression of Gastric Cancer by Targeting TIA1. Biochimie (2018) 154:119–26. doi: 10.1016/j.biochi.2018.08.006 30144499

[70] Liu y Liu R Yang F Cheng R Chen X Cui S. miR-19a Promotes Colorectal Cancer Proliferation and Migration by Targeting TIA1. Mol Cancer (2017) 16(1):53. doi: 10.1186/s12943-017-0625-8 28257633 PMC5336638

[71] Hamada j Shoda K Masuda K Fujita Y Naruto T Kohmoto T. Tumor-Promoting Function and Prognostic Significance of the RNA-Binding Protein T-Cell Intracellular Antigen-1 in Esophageal Squamous Cell Carcinoma. Oncotarget (2016) 7(13):17111–28. doi: 10.18632/oncotarget.7937 PMC4941375 26958940

[72] Tao Y Ma C Fan Q Wang Y Han T Sun C. MicroRNA-1296 Facilitates Proliferation, Migration And Invasion Of Colorectal Cancer Cells By Targeting SFPQ. J Cancer (2018) 9(13):2317–26. doi: 10.7150/jca.25427 PMC6036719 30026827

[73] de Silva HC Lin MZ Phillips L Martin JL Baxter RC. IGFBP-3 Interacts With NONO and SFPQ in PARP-Dependent DNA Damage Repair in Triple-Negative Breast Cancer. Cell Mol Life Sci: CMLS (2019) 76(10):2015–30. doi: 10.1007/s00018-019-03033-4 PMC11105386 30725116

