# Supplementary material for: Alternative Splicing-Based Differences Between Hepatocellular Carcinoma and Intrahepatic Cholangiocarcinoma: Genes, Immune Microenvironment, and Survival Prognosis
Source: Front Oncol. 2021 Oct 25;11:731993. doi: 10.3389/fonc.2021.731993 (PMC8574058; doi:10.3389/fonc.2021.731993)
Supplement: Supplementary file 2 [file DataSheet_2.docx]

Supplementary Material

# Supplementary Methods

**Functional Experiment of ADHFE1 Splicing Variants in HCC Cell lines and cell culture**

Liver cancer cell lines (HCCLM3, PLC/PRF/5, Hep3B, and Huh7) were purchased from the Shanghai Cell Collection (Shanghai, China). PLC/PRF/5 was cultured in Minimum Essential Medium with 10% FBS and 1% P/S; HCCLM3, Hep3B, and Huh7 cells were cultured in Dulbecco’s modified Eagle’s medium supplemented with 10% FBS and 1% P/S; and all cells were deposed in a humidified atmosphere with 5% CO2 at 37 °C.

**HCC tissue samples**

34 frozen HCC tissues and matched adjacent non-tumor liver tissues used in quantitative real-time PCR (qRT-PCR) were surgically resected from patients at the Affiliated Hospital of Qingdao University. Ethic permit was approved by local hospital ethic committee and informed consents were obtained from patients involved in the study.

**PCR RNA extraction and quantitative real-time PCR (qPCR)**

Total RNA was purified using TRIzol reagents (Shanghai Pufei Biotechnology). cDNA synthesis was performed using the Promega M-MLV Kit (Promega, USA). SYBR Premix EX Taq™ (TaKaRa, Otsu, Japan) was used for qPCR on a Real-Time PCR system (Roche LightCycler 480 II). Relative gene expression was determined by the comparative 2−ΔΔCT method. The primer sequences were as follows: ADHFE1_203-F: GGAGCAGCAGTTACAAAGGAAG; ADHFE1_203-R: CACCACCGACAGCAACATAGGC; ADHFE1_207-F: CCTCTGATTGCAGTGCCAACTAC; ADHFE1_207-R: GGATCAATCAGTCCCAGTGTGGG; GAPDH-F: TGACTTCAACAGCGACACCCA; GAPDH-R: CACCCTGTTGCTGTAGCCAAA.

**Lentivirus construction and infection of cell line**

LV_Vector, LV_ADHFE1_203 and LV_ADHFE1_207 lentiviruses were purchased from Genomeditech (Shanghai, China). Briefly, cells were infected with lentiviral particles in the presence of 5 μg/mL polybrene (Genomeditech, Shanghai, China); after 48 h, the supernatant was substituted with complete culture medium, and the transduced cells were selected for 7 days with 1 μg/mL puromycin (Solarbio, Beijing, China).

**MTT assay**

Cells were seeded in 96-well plates in triplicate at the initial density of 0.2 × 104 cells/well. At various time points, groups of cells were incubated with 20μl of 5 mg/ml sterile MTT [3-(4, 5-dimethyl-2-thiazolyl)-2, 5-diphenyl-2H-tetrazolium bromide; Genview] for 4 h at 37ºC, after which the culture medium was removed and 100μl of DMSO (Sigma, St. Louis, MO, USA) was added. The absorbance values were measured at 490 nm using 570 nm as the reference wavelength.

**Cell Cycle Analysis**

Harvested cells in a culture dish were fixed in 80% ice-cold ethanol in PBS after washing in ice-cold PBS. The cells were then pelleted in a cooled centrifuge and resuspended in cold PBS. Cells were incubated at 37°C for 30 min followed by the addition of bovine pancreatic RNAase (Sigma) at a final concentration of 2 mg/ml and 20 mg/ml of propidium iodide (Sigma-Aldrich) for 20 min at room temperature. Total 2x104 cells were analyzed using Guava easyCyte HT and data wasanalyzed using ModFit Software.

**Cell migration assay**

Cell migration was assessed using 8-μmpore transwell compartments (Corning, NY). For migration assays, 8×104 cells were suspended in serum-free medium in the upper compartment. After cells were incubated at 37 °C for 48 h, the translocated cells were stained with 0.5% crystal violet for 20 min at room temperature. For quantification, cells were counted under a light microscope (Nikon, Tokyo, Japan) in five fields (upper, lower, middle, left, and right; at × 200 magnification).

# Supplementary Figures

**
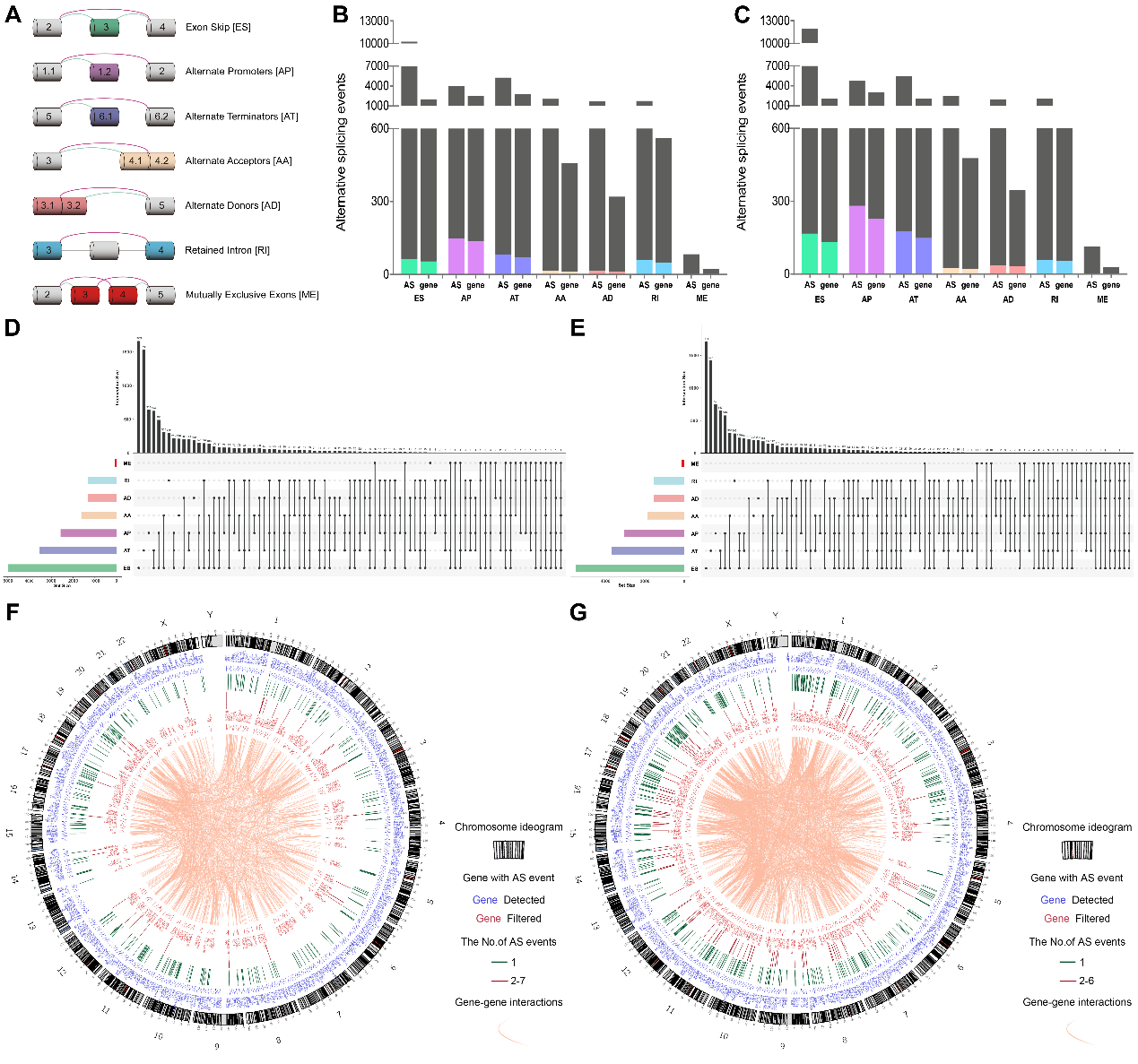
Supplementary Figure S1**. **Overview of AS events in HCC and ICC.** A, Illustration for splicing pattern of seven types of AS events. B and C, number of AS events and gene in HCC (B) and ICC (C), color bar represents the DEAS events and their parent genes. D and E, two UpSet plots show the interactive sets between seven types of AS events in HCC (D) and ICC (E), respectively. F and G, two plots were generated to show the details of AS events and the chromosome information of their corresponding genes in HCC patients (F) and ICC patients (G).


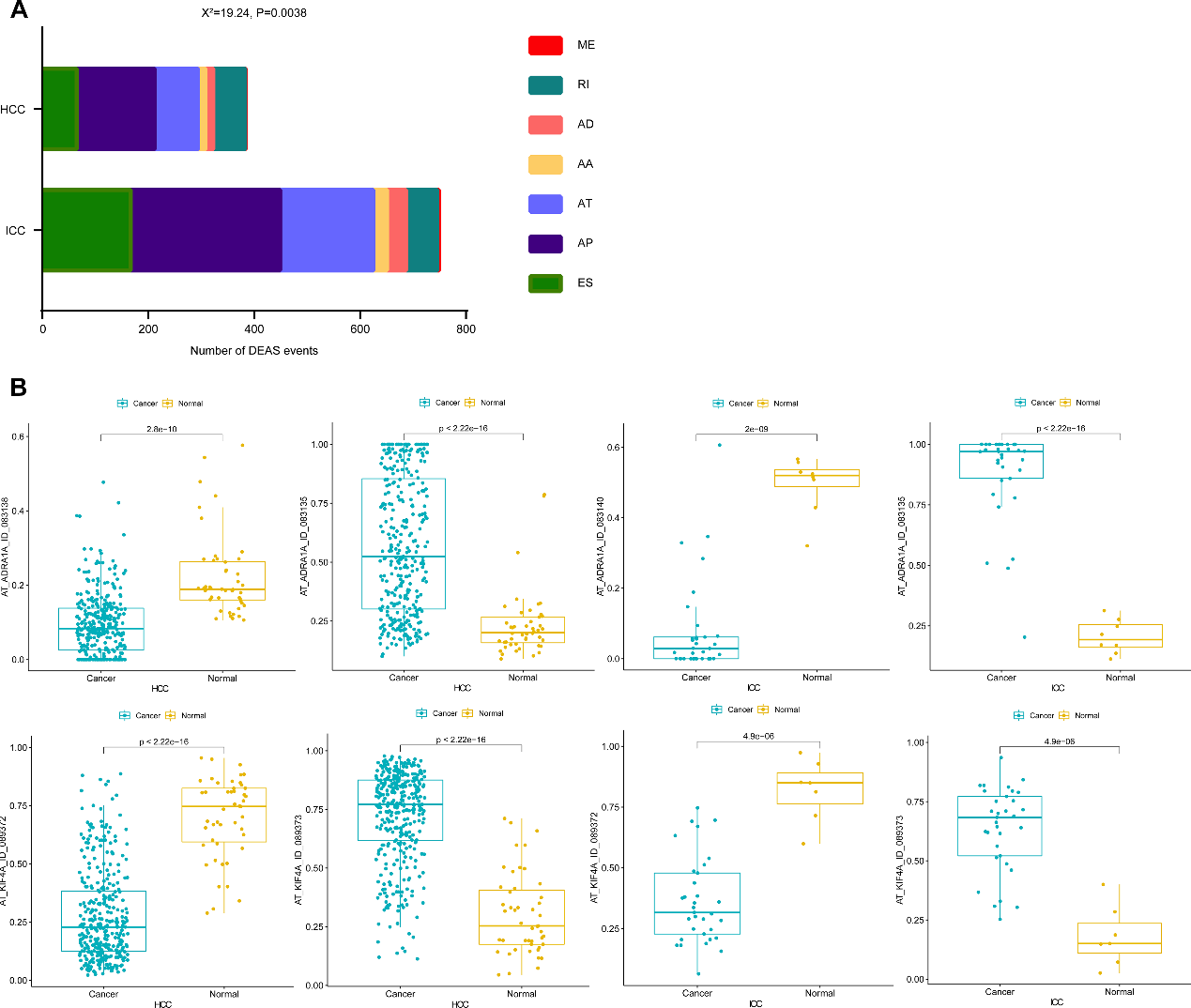


**Supplementary Figure S2, The difference analysis of DEAS events between HCC and ICC.** A, The difference analysis of seven splicing modes. B, several genes (such as ADRA1A and KIF4A) exhibited some opposite patterns of AS events in tumor and normal tissues.


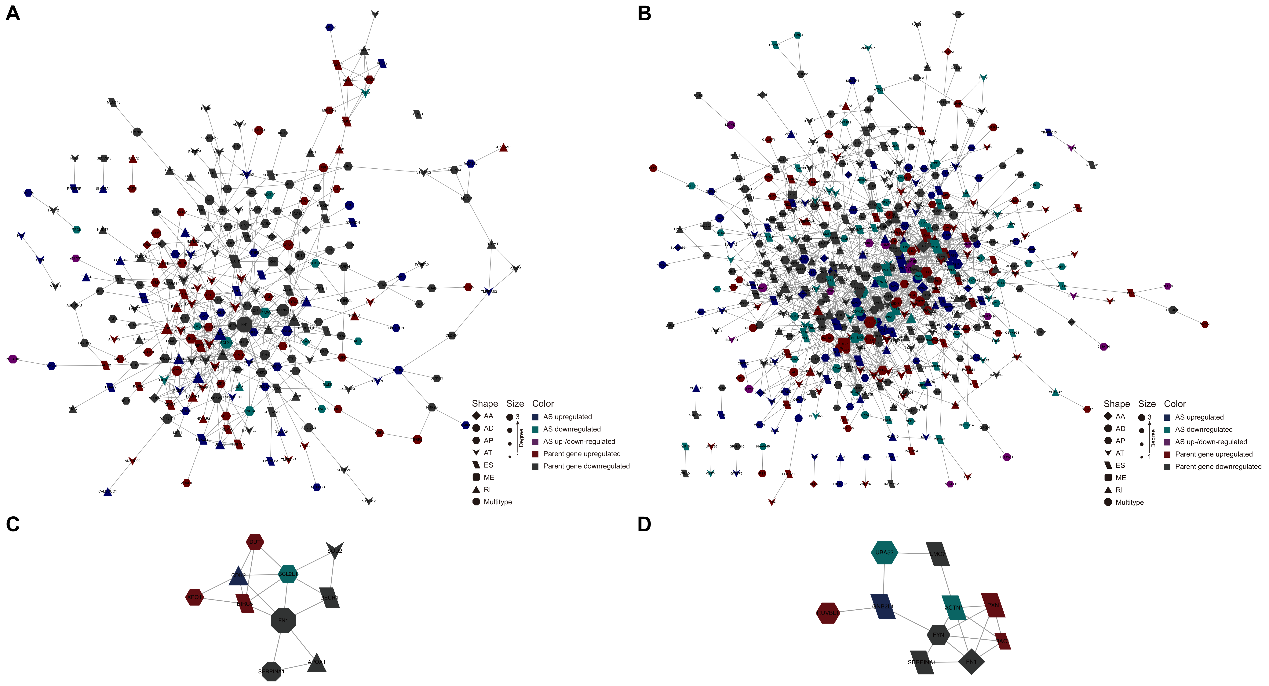


**Supplementary Figure S3, Protein-protein interaction analyses of DEAS events in HCC and ICC.** A, PPI analysis of DEAS events in HCC. Nodes represent parent genes with DEAS events. The shape, color and size of nodes represent splicing modes, change patterns and |log2FC|, respectively. Edges represent the potential interactions between the corresponding protein. B, PPI analysis of DEAS events in ICC. C-D, PPI analysis of the top 10 genes ranked by degree in HCC (C) and ICC (D).


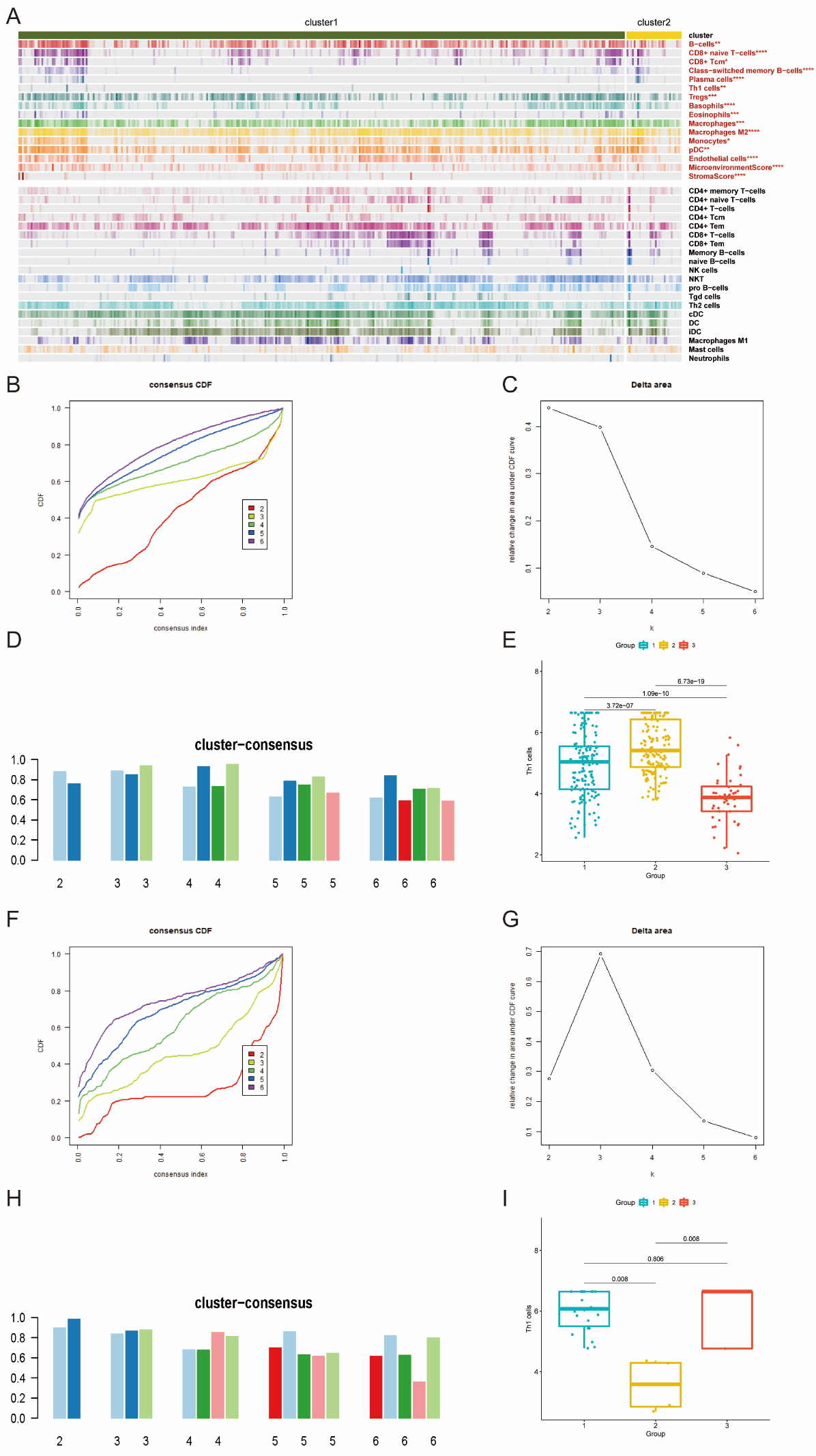


**Supplementary Figure S4, Immune features in HCC and ICC.** A, heatmap of the DEAS events in HCC and ICC, with annotations related with each cluster. B, CDF was used to determine the optimal number of clusters k in HCC (k=2 to 9). C, relative change in area under the CDF curve was used to determine the optimal number of clusters k in HCC (k=2 to 9). D, Cluster-consensus plot in HCC. E, the comparsion of one representative immune cell in 3 clusters. F, CDF was used to determine the optimal number of clusters k in ICC (k=2 to 9). G, relative change in area under the CDF curve was used to determine the optimal number of clusters k in ICC (k=2 to 9). H, Cluster-consensus plot in ICC. I, the comparsion of one representative immune cell in 3 clusters.


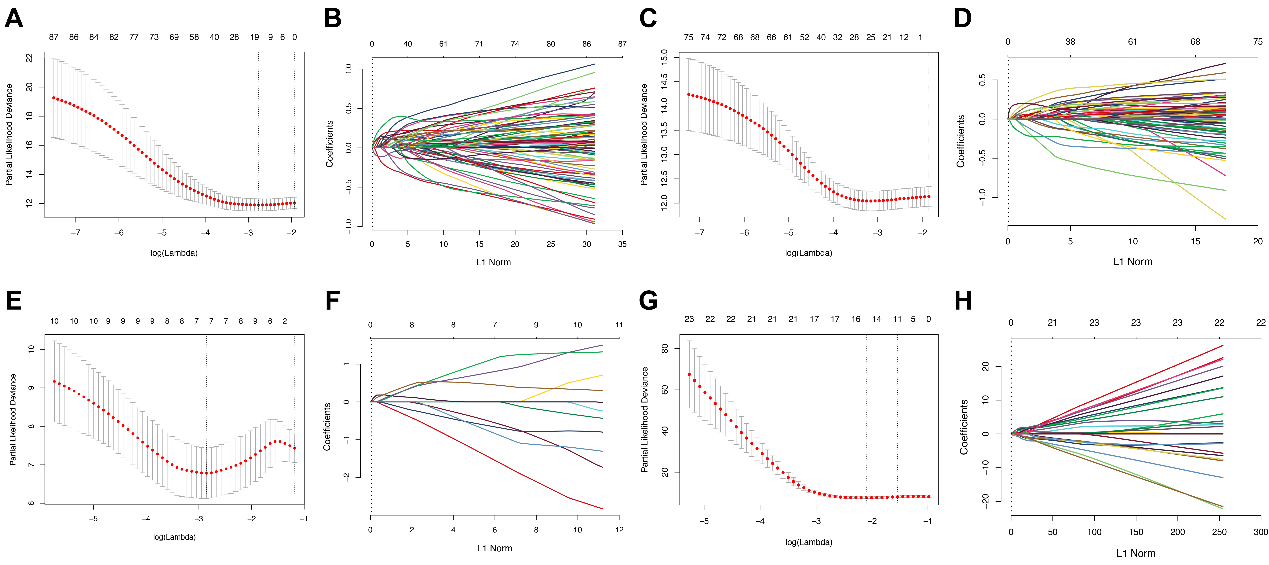


**Supplementary Figure S5**, **LASSO based on the meaningful DEAS events in univariate analysis in HCC and ICC.** A, partial likelihood deviance of the LASSO coefficient profiles for OS in HCC. B, each curve represents a meaningful DEAS event in univariate analysis of OS in HCC; ten‐fold cross‐validation was used to calculate best lambda which leads to minimum mean cross‐validated error. C, partial likelihood deviance of the LASSO coefficient profiles for DFS in HCC. D, each curve represents a meaningful DEAS event in univariate analysis of DFS in HCC; ten‐fold cross‐validation was used to calculate best lambda which leads to minimum mean cross‐validated error. E, partial likelihood deviance of the LASSO coefficient profiles for OS in ICC. F, each curve represents a meaningful DEAS event in univariate analysis of OS in ICC; ten‐fold cross‐validation was used to calculate best lambda which leads to minimum mean cross‐validated error. G, partial likelihood deviance of the LASSO coefficient profiles for DFS in ICC. H, each curve represents a meaningful DEAS event in univariate analysis of DFS in ICC; ten‐fold cross‐validation was used to calculate best lambda which leads to minimum mean cross‐validated error.


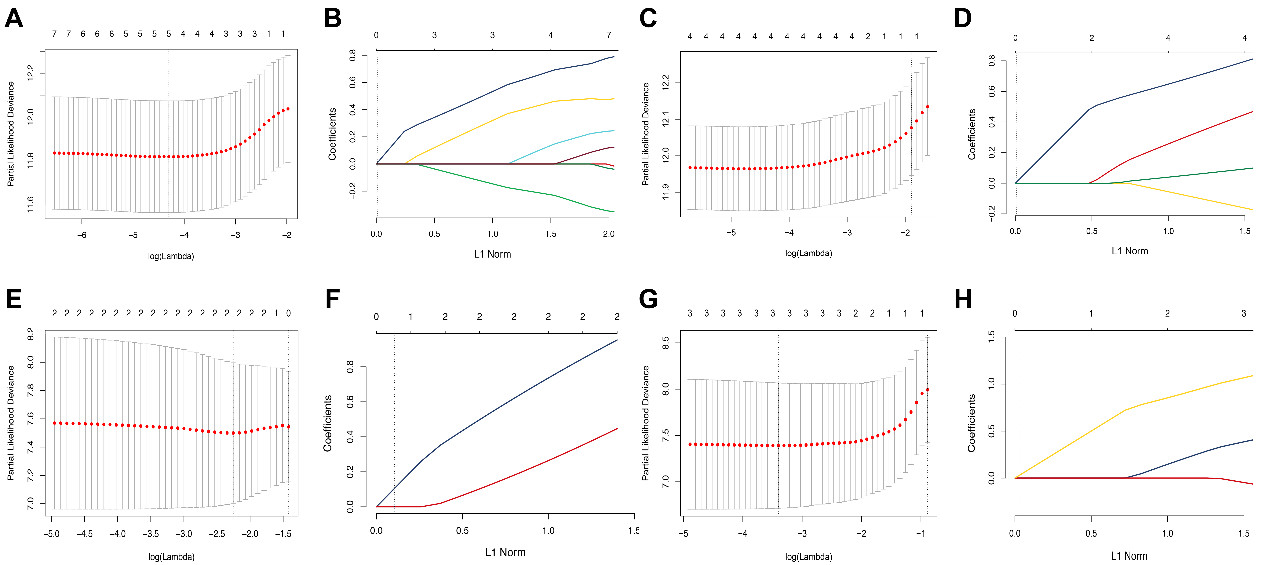


**Supplementary Figure S6,** **LASSO based on the DEAS models and** **clinicopathological data in HCC and ICC.** A, partial likelihood deviance of the LASSO coefficient profiles for OS in HCC. B, each curve represents an OS-variable in HCC; ten‐fold cross‐validation was used to calculate best lambda which leads to minimum mean cross‐validated error. C, partial likelihood deviance of the LASSO coefficient profiles for DFS in HCC. D, each curve represents a DFS-variable in HCC; ten‐fold cross‐validation was used to calculate best lambda which leads to minimum mean cross‐validated error. E, partial likelihood deviance of the LASSO coefficient profiles for OS in ICC. F, each curve represents an OS-variable in ICC; ten‐fold cross‐validation was used to calculate best lambda which leads to minimum mean cross‐validated error. G, partial likelihood deviance of the LASSO coefficient profiles for DFS in ICC. H, each curve represents a DFS-variable in ICC; ten‐fold cross‐validation was used to calculate best lambda which leads to minimum mean cross‐validated error.


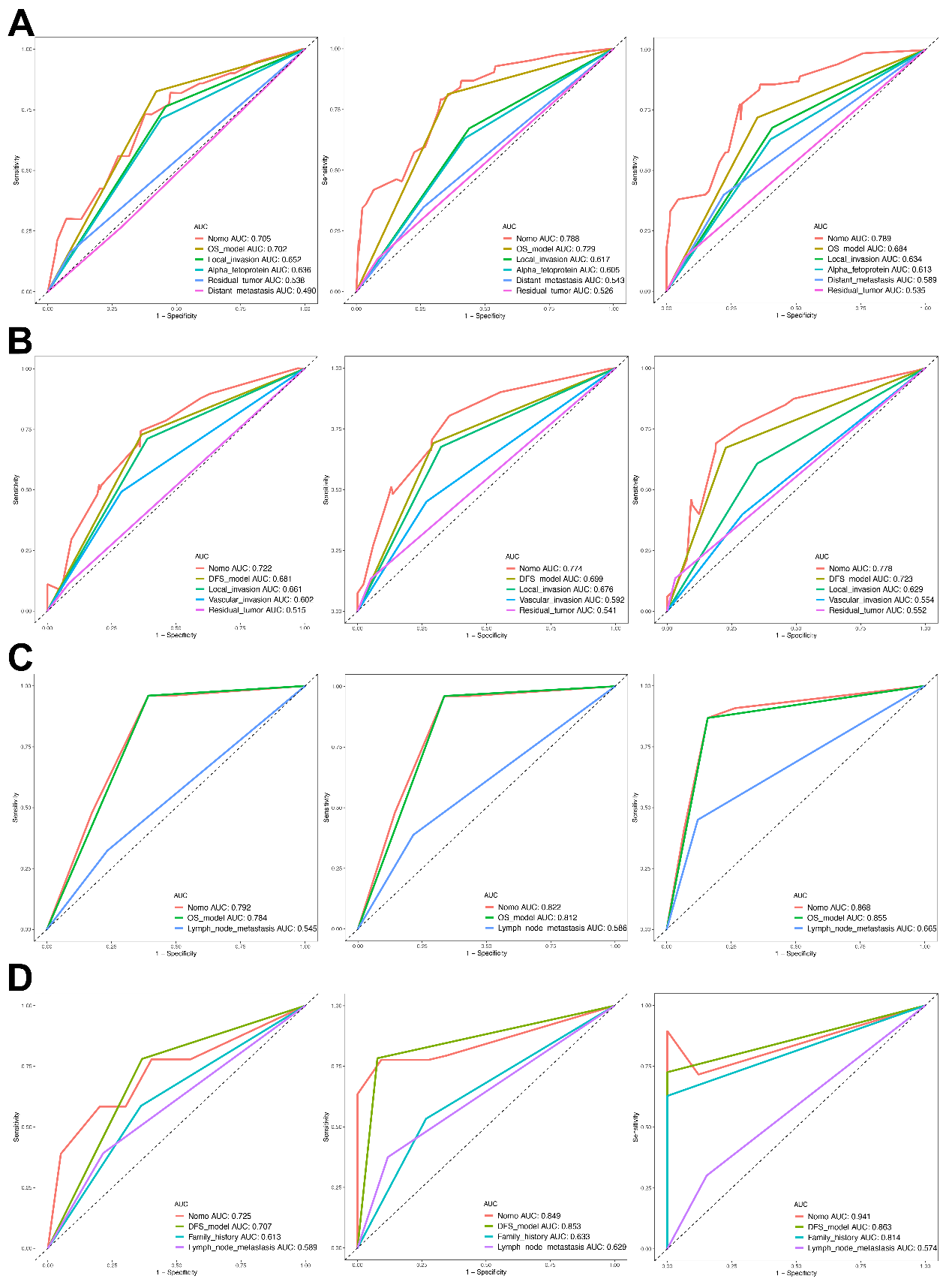


**Supplementary Figure S7**, **The ROC curves of Nomogram models in HCC and ICC.** A-B, the ROC curves of nomogram to predict the 1, 2 and 3-years OS (A) and DFS (B) in HCC. C-D, the ROC curves of nomogram to predict the 0.5, 1 and 2-years OS (C) and DFS (D) in ICC.


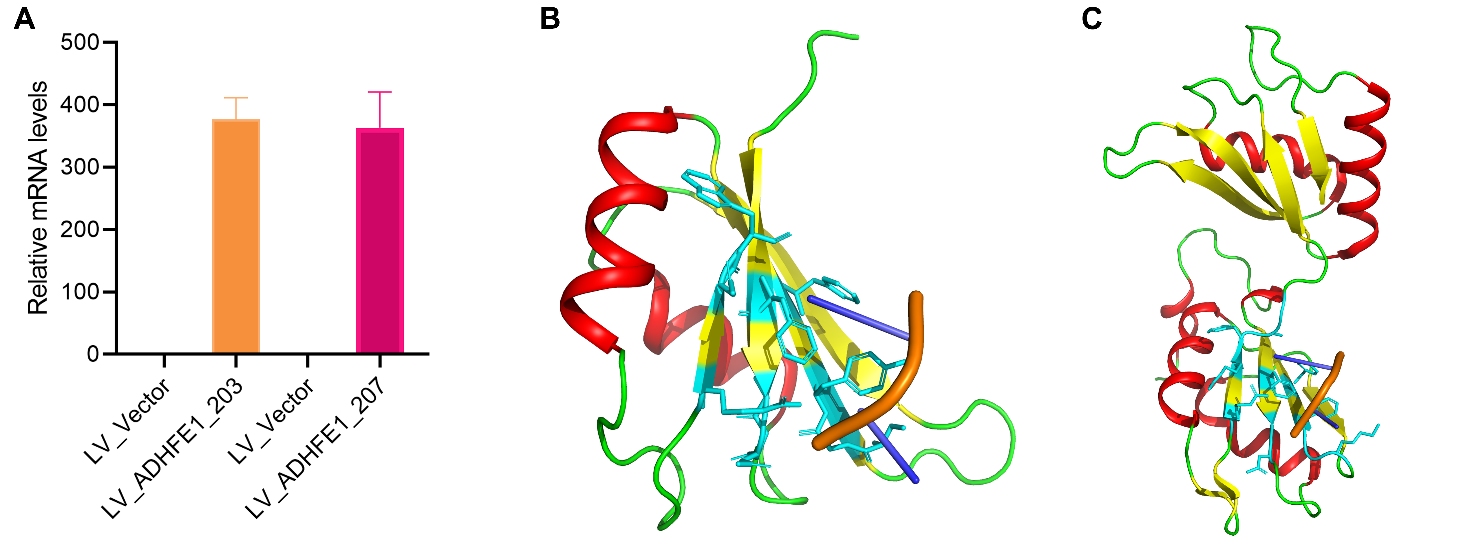


**Supplementary Figure S8, qPCR results of the overexpression and the protein crystal structure of TIA1 and SFPQ.** A**,** qPCR results of the overexpression. B, crystal structure of TIA1 RNA Recognition Motif 2 (RRM2) domain (PDB 5O3J) bound with a single chain RNA molecule. RRM2 domain shown in cartoon, beta sheet colored in yellow, protein domain that binds to RNA is colored in light blue, helices in red, loops in green. RNA backbone is shown as brown ribbon, bases as blue stick. C, crystal structure of SFPQ RRM1 domain (PDB 6NCQ) bound to a modeled RNA chain.
